# Maternal, fetal and neonatal outcomes among pregnant women receiving COVID-19 vaccination: The preg-co-vax study

**DOI:** 10.3389/fimmu.2022.965171

**Published:** 2022-10-03

**Authors:** Annamaria Mascolo, Gabriella di Mauro, Federica Fraenza, Mario Gaio, Alessia Zinzi, Ciro Pentella, Francesco Rossi, Annalisa Capuano, Liberata Sportiello

**Affiliations:** ^1^ Campania Regional Centre for Pharmacovigilance and Pharmacoepidemiology, University of Campania “Luigi Vanvitelli”, Naples, Italy; ^2^ Department of Experimental Medicine, University of Campania “Luigi Vanvitelli”, Naples, Italy; ^3^ UOC Pharmacy, AORN Santobono-Pausilipon Children’s Hospital, Naples, Italy

**Keywords:** pregnant women, COVID-19 vaccines, maternal exposure during pregnancy, adverse events following immunization, pharmacovigilance, database

## Abstract

**Introduction:**

Although the European Medicines Agency (EMA) encourage coronavirus disease 2019 (COVID-19) vaccination in pregnant women, the scientific evidence supporting the use of COVID-19 vaccines during pregnancy is still limited.

**Aim:**

We aimed to investigate adverse events following immunization (AEFI) with COVID-19 vaccines during pregnancy.

**Methods:**

We retrieved Individual Case Safety Reports (ICSRs) related to the use of COVID-19 vaccines during pregnancy from the EudraVigilance database for the year 2021. We analyzed AEFI related to the mother and fetus/newborn. The reporting odds ratio (ROR) was computed to compare the reporting probability of spontaneous abortion between COVID-19 vaccines.

**Results:**

During the study period, among 1,315,315 ICSRs related to COVID-19 vaccines, we retrieved 3,252 (0.25%) reports related to the use in pregnancy. More than half (58.24%) of ICSRs were submitted by non-healthcare professionals. Although the majority (87.82%) of ICSRs concerned serious AEFI, their outcomes were mostly favorable. In this study, 85.0% of total ICSRs referred to pregnant women (n = 2,764), while 7.9% referred to fetuses/newborns (n = 258). We identified 16,569 AEFI. Moreover, 55.16% were AEFI not related to pregnancy (mostly headache, pyrexia, and fatigue), while 17.92% were pregnancy-, newborn-, or fetus-related AEFI. Among pregnancy-related AEFI, the most reported was spontaneous abortion. Messenger RNA (mRNA) vaccines had a lower reporting probability of spontaneous abortion than viral vector-based vaccines (ROR 0.80, 95% CI 0.69–0.93). Moderna and Oxford-AstraZeneca vaccines had a higher reporting probability of spontaneous abortion (ROR 1.2, 95% CI 1.05–1.38 and ROR 1.26, 95% CI 1.08–1.47, respectively), while a lower reporting probability was found for Pfizer-BioNTech vaccine compared with all other COVID-19 vaccines (ROR 0.73, 95% CI 0.64–0.84). In addition, 5.8% of ICSRs reported a fatal outcome.

**Conclusions:**

No strong insight of unknown AEFI associated with COVID-19 vaccination in pregnant women was observed. Considering the high risk associated with severe acute respiratory syndrome coronavirus 2 (SARS-CoV-2) infection, our analysis suggests that the benefits of COVID-19 vaccines during pregnancy outweigh the possible risks. However, it is important to continue monitoring the safety profile of COVID-19 vaccines in this subpopulation.

## Introduction

The rapid emergence and human-to-human transmission of the severe acute respiratory syndrome coronavirus 2 (SARS-CoV-2) resulted in the global pandemic of coronavirus disease 2019 (COVID-19) associated with considerable morbidity and mortality. This led to the need of using innovative techniques to rapidly develop new vaccines to prevent COVID-19. To date, the European Medicines Agency (EMA) approved five COVID-19 vaccines, of which two are messenger RNA (mRNA) vaccines (Pfizer-BioNTech and Moderna), two are viral vector-based vaccines (Oxford-AstraZeneca and Janssen), and one is a protein subunit vaccine (Novavax) (1). None of the COVID-19 vaccines approved under the conditional marketing authorization was tested in pregnant women during the initial trials (2). This “knowledge gap” is typical of pre-marketing clinical trials, which do not involve pregnant women. Consequently, as for any other drug, pregnancy-related data on the use of these vaccines can be obtained only after marketing approval.

With regard to their safety profile, the observational study conducted by Shimabukuro et al. (3), using preliminary data from the “v-safe after vaccination health checker” surveillance system, the v-safe pregnancy registry, and the Vaccine Adverse Event Reporting System (VAERS), did not show any safety signals among pregnant women who received mRNA COVID-19 vaccination. Other observational data from pregnant women vaccinated with mRNA COVID-19 vaccines during the second and third trimester did not show an increase in adverse pregnancy outcomes (4, 5). Instead, data on pregnancy outcomes following mRNA COVID-19 vaccination during the first trimester are insufficient, and no correlation with the risk of miscarriage emerged. Limited data are available on the safety of viral vector-based COVID-19 vaccines during pregnancy, though adenovirus vector-based vaccines have been tested in pregnant animal models without safety concerns (6, 7). Based on these data, the EMA considered the mRNA COVID-19 vaccination during pregnancy to be safe, while viral vector COVID-19 vaccines are to be used only after a close consultation with a healthcare professional (HCP) for weighing benefits and risks. Among safety issues, spontaneous abortion is a priority safety outcome in studies of maternal exposure to vaccines (8), and women are alarmed for this risk as much to hesitate to get vaccinated during pregnancy (9).

Although the EMA supports and encourages mRNA COVID-19 vaccination in this population, the scientific evidence is still limited, and further studies are needed to monitor the safety profile of COVID-19 vaccines in pregnant women. In light of this, by using one of the largest pharmacovigilance databases, i.e., EudraVigilance (EV), we carried out an analysis of Individual Case Safety Reports (ICSRs) of adverse events following immunization (AEFI) with COVID-19 vaccines during pregnancy, providing important insights on their safety profile.

## Methods

### Data source

The EV is the European database designed for collecting ICSRs of suspected adverse drug reactions (ADRs) and AEFI, developed and maintained by the EMA since December 2001. These reports are used for evaluating the benefits and risks of medicines and vaccines during their development and for monitoring their safety after authorization. In 2012, EMA launched a website (http://www.adrreports.eu/en/index.html) to provide public access to ICSRs, submitted electronically to EV. In this way, all stakeholders involved in pharmacovigilance, including the general public, can access aggregated safety information used by European regulatory authorities. From the website, ICSRs are retrieved for a single drug or vaccine and reported in a unique Excel file. ICSRs contain the following information: patient sex [female, male, not specified], two age groups for patient [1 group: 0–1 month, 2 months–2 years, 3–11 years, 12–17 years, 18–64 years, 65–85 years, more than 85 years; 2 group (as reporter): fetus, neonate, infant, child, adolescent, adult, elderly, not specified], primary source qualification (HCP, non-HCP), geographic origin [European Economic Area (EEA), non-EEA], adverse reaction list, suspect/interacting and concomitant drug list, outcome (recovered/resolved, recovering/resolving, not recovered/not resolved, fatal, recovered/resolved with sequelae, unknown), and seriousness (results in death, life threatening, congenital anomaly, disabling, caused/prolonged hospitalization, other medically important condition). The seriousness criterion “Other Medically Important Condition” includes any AEFI not covered by other criteria and considered by a reporter as serious or any AEFI contained in the Important Medical Event list of the EMA. This list aims to facilitate the classification of AEFI/ADRs for pharmacovigilance activities in the EU (10). Signs and symptoms of AEFI/ADR in ICSRs are coded according to the preferred terms (PTs) of Medical Dictionary for Regulatory Activities (MedDRA), the internationally standardized medical terminology. MedDRA is a hierarchical system starting with a very general level (the system organ classes) and ending with the more detailed level (PTs), which is in turn divided into the most specific level, namely, low-level term (LLT) (11). Other MedDRA details are described elsewhere (12–14). An ICSR can report one or more MedDRA PTs related to one or more drugs/vaccines.

### Data retrieval

On 27 January 2022, we searched the EV website (www.adrreport.eu) for all ICSRs related to the four COVID-19 vaccines [Pfizer-BioNTech, Moderna, Oxford-AstraZeneca, and Janssen] by using the line-listing function for the period from 1 January 2021 to 31 December 2021. The Novavax vaccine was not considered because it was authorized later (20 December 2021). To identify pregnancy-related ICSRs (also defined as cases), we performed an approach based on three steps. Firstly, we obtained the preliminary dataset by conducting an automated selection using a text string search for the term “pregnancy” in the column related to the PT list and the term “transplacental” in the column related to the route of vaccine administration. We also retrieved ICSRs with women exposed to COVID-19 vaccines during pregnancy when the AEFI being reported (or the focus of the report) was in the born neonate/infant or was reported fetus, neonate, or infant as age group. Secondly, from this group of ICSRs, we extracted the intermediate dataset including ICSRs with a plausible temporal correlation between exposure to COVID-19 vaccines and the pregnancy status. To achieve this aim, we selected as extraction criteria any of the following PTs: “Drug exposure during pregnancy,” “Drug exposure in pregnancy,” “Exposure during pregnancy,” “Fetal exposure during pregnancy,” “Maternal exposure during pregnancy,” and “Maternal exposure timing unspecified.” Thirdly, to get the final dataset, we excluded ICSRs with PTs referred to extraction criteria and without AEFI, the uncertain information on the vaccine exposure during pregnancy, and sex or age implausible/incoherent or unknown (e.g., male adult, women with age >65 years). ICSRs were classified according to the suspected COVID-19 vaccines, and cases reporting two different COVID-19 vaccine products were grouped as “mix vaccination.”

### Data analyses

After the selection of ICSRs, we analyzed them for each COVID-19 vaccine (Pfizer-BioNTech, Moderna, Oxford-AstraZeneca, and Janssen). ICSRs were categorized for gender, age, primary source qualification, primary source country for regulatory purposes, number of suspected or concomitant drugs, seriousness, and outcome. In each category, we indicated “not specified” if the information was not provided. Moreover, we categorized for each COVID-19 vaccine the AEFI according to the Standardized MedDRA Queries (SMQs): 1) “Congenital, familial, and genetic disorders”; 2) “Pregnancy, labor, and delivery complications and risk factors (excluding abortions and stillbirth)”; 3) “Neonatal disorders”; 4) “Termination of pregnancy and risk of abortion”; and 5) “Fetal disorders”; all members of the SMQ “Pregnancy and neonatal topics.” All PTs not reported in these SMQs were classified in the following: 6) “Maternal other AEFI” if they occurred in pregnant women and 7) “Fetal other AEFI” and 8) “Neonatal other AEFI” if they occurred in fetus or born neonate/infant, respectively. All other PTs not included in the aforementioned groups and not indicative of AEFI were reported as 9) “PT not indicating AEFI.” All maternal and fetal/neonatal AEFI classified in these nine groups were tabled for each COVID-19 vaccine. The five most reported PTs for each event group were estimated on the total number of PTs and then distributed for each COVID-19 vaccine.

Fatal ICSRs associated with COVID-19 vaccines, except for abortion, were evaluated by pointing out the most clinically relevant events. Moreover, ICSRs with an AEFI related to death but without the outcome or seriousness reported as “fatal/results in death” were reclassified as fatal. All data analyses were performed using Excel (Microsoft Excel, Excel version 2021) and the statistical software R Studio (version 3.2.2, R Development Core Team).

### Disproportionality analyses

Among the five most frequently reported PTs for each pregnancy-related Event Group (except “Maternal other AEFI”), only if a PT was reported for all COVID-19 vaccines and in a number of cases ≥3, we also performed a disproportionality analysis to assess the reporting frequency of that PT for mRNA vaccines vs. viral vector-based vaccines, each COVID-19 vaccine compared to all other COVID-19 vaccines, and Moderna vs. Pfizer-BioNTech. Therefore, the reporting odds ratio (ROR), its 95% confidence interval (95% CI), and the chi-square test were computed. The ROR was calculated as (a/c)/(b/d): “a” is the number of a specific PT notified with the selected vaccine/grouped vaccines, “c” the number of a specific PT notified with the comparator, “b” the number of extra-specific PTs notified with the selected vaccine/grouped vaccines, and “d” the number of extra-specific PTs notified with comparator. The P value <0.05 was used for statistical significance.

### Compliance with ethical standards

Safety data deriving from the spontaneous reporting system are anonymous and follow ethical standards; therefore, no further ethical measure was required.

## Results

**Figure 1:**
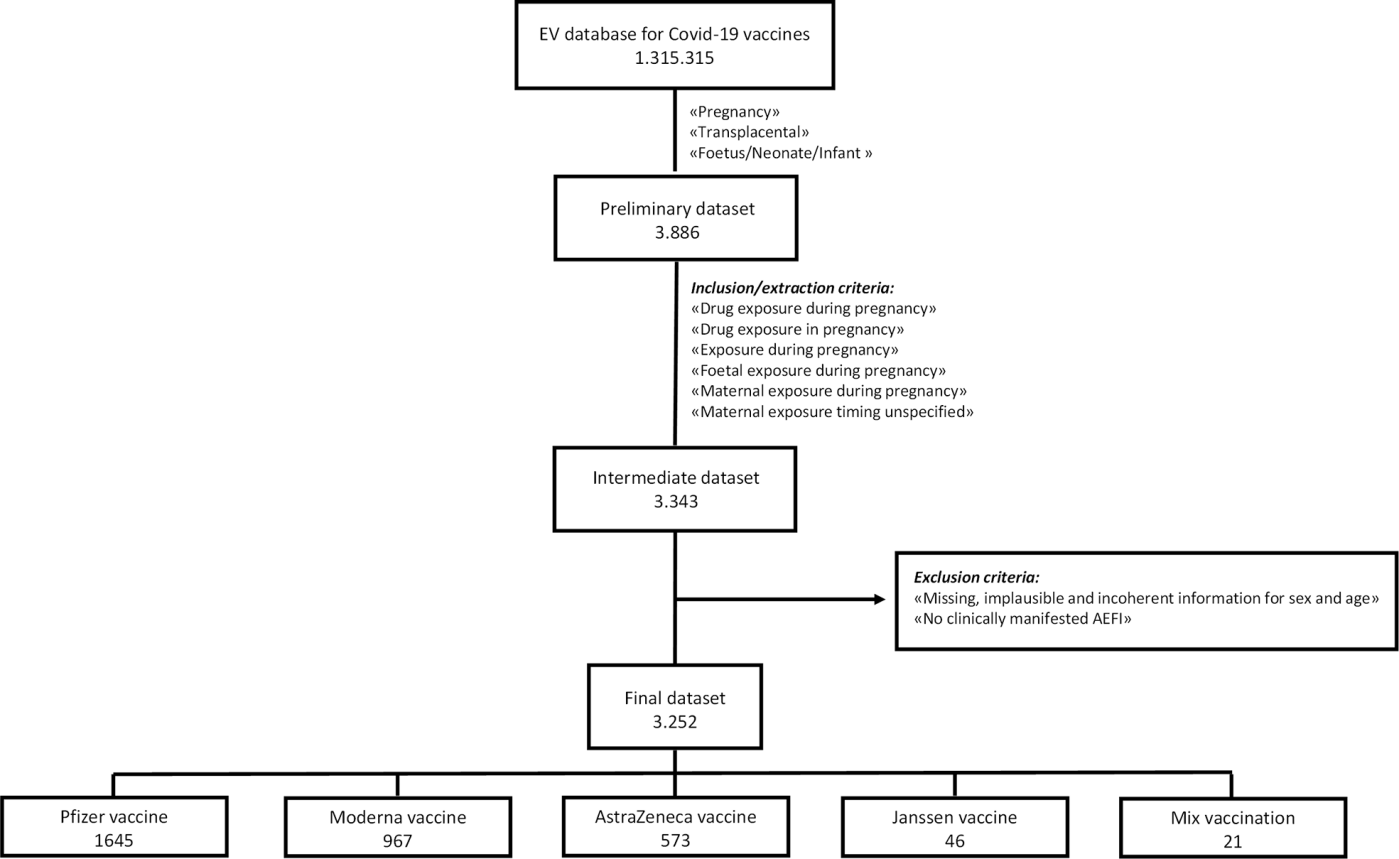
Flowchart of the selection process of ICSRs from the EudraVigilance database. AEFI, adverse event following immunization; EV, Eudravigilance.

In the study period, 1,315,315 ICSRs related to the four COVID-19 vaccines were retrieved from EV: 659,656 for Pfizer-BioNTech, 186,486 for Moderna, 426,864 for Oxford-AstraZeneca, and 42,309 for Janssen vaccine. As shown in the flowchart (**Figure 1**), our preliminary dataset contained 3,886 ICSRs (1,711 for Pfizer-BioNTech, 1,228 for Moderna, 868 for Oxford-AstraZeneca, and 79 for Janssen vaccine). A total of 3,343 out of 3,886 cases fulfilled the data extraction criteria (1,659 for Pfizer-BioNTech, 1,014 for Moderna, 621 for Oxford-AstraZeneca, and 49 for Janssen vaccine). From this intermediate dataset, we excluded 91 ICSRs for the following reasons: male sex, not specified or incoherent age, and cases without clinically manifested AEFI.

Overall, 3,252 cases were included, of which 1,645 (50.6%) referred to Pfizer-BioNTech vaccine, 967 (29.7%) to Moderna vaccine, 573 (17.6%) to Oxford-AstraZeneca vaccine, 46 (1.4%) to Janssen vaccine, and 21 (0.6%) to mix vaccination (12 for the combination Oxford-AstraZeneca/Pfizer-BioNTech, five for the combination Moderna/Pfizer-BioNTech, and four for the combination Moderna/Oxford-AstraZeneca). Most cases were related to events that occurred in pregnant women (n = 2,764; 85.0%) than those that occurred in child age groups (n = 258; 7.9%). As expected, 83.76% (n = 2,724) of pregnant women were aged 18–64 years; only 11 (0.34%) cases involved adolescent pregnant women aged 12–17 years. The mean number of events per ICSR was 3.1. Regarding distribution by seriousness, 87.82% (n = 2,856) of ICSRs reported AEFI that were considered serious, while 12.18% (n = 396) reported AEFI that were classified as not serious. Similar distributions were observed for each COVID-19 vaccine.

The main primary source was non-HCP for ICSRs with Pfizer-BioNTech (1,162; 70.64%), Oxford-AstraZeneca (291; 50.79%), Janssen (25; 54.35%) vaccines, and mix vaccination (13; 61.90%), while it was HCP for Moderna (564; 58.32%) vaccine. The majority of all ICSRs (2,275; 69.96%) were from the non-EEA, while the remaining were from the EEA. In almost all ICSRs (97.66%), the vaccine was the only suspect reported. In slightly more than half of cases (n = 1,686; 51.85%), concomitant drugs were not reported and, if present, there was mostly one concomitant drug (n = 913; 28.08%). All characteristics of ICSRs for type of COVID-19 vaccine are reported in **Table 1**.

**Table 1:** Distribution for age group, seriousness, primary source, primary source country for regulatory purposes, presence of other suspected or concomitant drugs among Individual Case Safety Reports (ICSRs) related to the maternal exposure during pregnancy to COVID-19 vaccines reported in EudraVigilance from 1 January 2021 to 31 December 2021.

Characteristics	Level	mRNA vaccines		Viral vector-based vaccines		Mix vaccination*(n=21)	Total(n=3,252)
		Pfizer(n=1,645)	Moderna(n=967)	AstraZeneca(n=573)	Janssen(n=46)		
**Number of events per ICSR**	*Mean*	2.8	3.2	3.5	3.9	3.9	3.1
**Mother Age Group**	*12-17 years*	7 (0.43)	2 (0.21)	2 (0.35)	0 (0.00)	0 (0.00)	11 (0.34)
	*18-64 years*	1,273 (77.39)	884 (91.42)	512 (89.35)	37 (80.43)	18 (85.71)	2,724 (83.76)
	*Adult age Not Specified*	20 (1.22)	2 (0.21)	7 (1.22)	0 (0.00)	0 (0.00)	29 (0.89)
**Child Age Group**	*Fetus*	158 (9.60)	4 (0.41)	1 (0.17)	0 (0.00)	0 (0.00)	163 (5.01)
	*Neonate (0 – 1m)*	22 (1.34)	7 (0.72)	4 (0.70)	1 (2.17)	2 (9.52)	36 (1.11)
	*Infant (2m – 2y)*	10 (0.61)	10 (1.03)	2 (0.35)	1 (2.17)	0 (0.00)	23 (0.71)
	*Neonatal age Not Specified*	27 (1.64)	4 (0.41)	5 (0.87)	0 (0.00)	0 (0.00)	36 (1.11)
**Not Specified Age Group**	*Not specified*	128 (7.78)	54 (5.58)	40 (6.98)	7 (15.22)	1 (4.76)	230 (7.07)
**Seriousness of ICSR**	*Serious (%)*	1,443 (87.72)	874 (90.38)	477 (83.25)	43 (93.48)	19 (90.48)	2,856 (87.82)
	*Not serious (%)*	202 (12.28)	93 (9.62)	96 (16.75)	3 (6.52)	2 (9.52)	396 (12.18)
**Primary Source**	*Healthcare Professional (%)*	483 (29.36)	564 (58.32)	282 (49.21)	21 (45.65)	8 (38.10)	1,358 (41.76)
	*Non-Healthcare Professional (%)*	1,162 (70.64)	403 (41.68)	291 (50.79)	25 (54.35)	13 (61.90)	1,894 (58.24)
**Primary Source Country for Regulatory Purposes**	*European Economic Area (%)*	692 (42.07)	176 (18.20)	93 (16.23)	13 (28.26)	3 (14.29)	977 (30.04)
	*Non-European Economic Area (%)*	953 (57.93)	791 (81.80)	480 (83.77)	33 (71.74)	18 (85.71)	2,275 (69.96)
**Other suspected drug(s)**	*0 (%)* *1 (%)* *2 (%)* *3 (%)* ≥4 *(%)*	1,620 (98.48) 21 (1.28) 3 (0.18) 1 (0.06) 0 (0.00)	957 (98.97) 10 (1.03) 0 (0.00) 0 (0.00) 0 (0.00)	551 (96.16) 20 (3.49) 1 (0.17) 1 (0.17) 0 (0.00)	45 (97.83) 1 (2.17) 0 (0.00) 0 (0.00) 0 (0.00)	3 (14.29) 18 (85.71) 0 (0.00) 0 (0.00) 0 (0.00)	3,176 (97.66) 70 (2.15) 4 (0.12) 2 (0.06) 0 (0.00)
**Concomitant drug(s)**	*0 (%)* *1 (%)* *2 (%)* *3 (%)* ≥*4 (%)*	946 (57.51) 432 (26.26) 135 (8.21) 62 (3.77) 70 (4.25)	454 (46.95) 272 (28.13) 115 (11.89) 53 (5.48) 73 (7.55)	249 (43.46) 192 (33.51) 67 (11.69) 24 (4.19) 41 (7.15)	31 (67.39) 9 (19.57) 0 (0.00) 3 (6.52) 3 (6.52)	6 (28.57) 8 (38.10) 3 (14.29) 0 (0.00) 4 (19.05)	1,686 (51.85) 913 (28.08) 320 (9.84) 142 (4.37) 191 (5.87)

* Mix vaccination was represented by the following combinations: Oxford-AstraZeneca/Pfizer-BioNTech, Moderna/Pfizer-BioNTech, and Moderna/Oxford-AstraZeneca.

ICSRs, Individual Case Safety Reports; mRNA, messenger RNA.

Data are expressed as n (%).

### Adverse event following immunization (AEFI) for maternal exposure during pregnancy to COVID-19 vaccines

All ICSRs (n = 3,252) were associated with 13,659 PTs, of which 6,429 were for Pfizer-BioNTech, 4,165 for Moderna, 2,691 for Oxford-AstraZeneca, 253 for Janssen vaccine, and 121 for mix vaccination (**Table 2**). A total of 7,534 (55.16%) involved non-pregnancy-specific adverse events (Event Group “Maternal other AEFI”), while 2,448 (17.92%) involved pregnancy-, neonatal-, or fetal-specific adverse events (all other Event Groups except for “PTs not of interest”).

**Table 2:** AEFI related to maternal exposure during pregnancy to COVID-19 vaccines distributed by Event Groups and Preferred Terms§.

Event Groups	mRNA vaccines		Viral vector-based vaccines		Mix vaccination*(n=121)	*Total*(n=13,659)
		Pfizer(n=6,429)	Moderna(n=4,165)	AstraZeneca(n=2,691)			Janssen(n=253)	
**Maternal other AEFI** Headache Pyrexia Fatigue Myalgia Pain in extremity	**3,389 (52.71)** 211 (3.28) 126 (1.96) 173 (2.69) 98 (1.52) 110 (1.71)	**2,287 (54.91)** 122 (2.93) 133 (3.19) 136 (3.27) 71 (1.70) 93 (2.23)	**1,660 (61.69)** 139 (5.17) 145 (5.39) 79 (2.94) 91 (3.38) 31 (1.15)	**139 (54.94)** 4 (1.58) 8 (3.16) 6 (2.37) 2 (0.79) 2 (0.79)	**59 (48.86)** 4 (3.31) 2 (1.65) 8 (6.61) 2 (1.65) 6 (4.96)	**7,534 (55.16)** 480 (3.51) 414 (3.03) 402 (2.94) 264 (1.93) 242 (1.77)
**Termination of pregnancy and risk of abortion** Abortion spontaneous Fetal death Abortion missed Stillbirth Abortion	**490 (7.62)** 381 (5.93) 41 (0.64) 35 (0.54) 8 (0.12) 5 (0.08)	**428 (10.28)** 326 (7.83) 45 (1.08) 14 (0.34) 15 (0.36) 5 (0.12)	**270 (10.03)** 222 (8.25) 2 (0.07) 5 (0.19) 4 (0.15) 17 (0.63)	**20 (7.91)** 19 (7.51) - - 1 (0.40) -	**6 (4.96)** 4 (3.31) 1 (0.83) 1 (0.83) - -	**1,214 (8.89)** 952 (6.97) 89 (0.65) 55 (0.40) 28 (0.20) 27 (0.20)
**Pregnancy, labor, and delivery complications and risk factors (excluding abortions and stillbirth)** Hemorrhage in pregnancy Uterine contractions during pregnancy Premature labor Premature delivery Preeclampsia	**255 (3.97)** 35 (0.54) 44 (0.68) 24 (0.37) 11 (0.17) 6 (0.09)	**249 (5.98)** 31 (0.74) 13 (0.31) 26 (0.62) 23 (0.55) 16 (0.38)	**52 (1.93)** 2 (0.07) 1 (0.04) 5 (0.19) 3 (0.11) 5 (0.19)	**12 (4.74)** - - 1 (0.40) 2 (0.79) 1 (0.40)	**7 (5.79)** 1 (0.83) - - 2 (1.65) -	**575 (4.21)** 69 (0.51) 58 (0.42) 56 (0.41) 41 (0.30) 28 (0.20)
**Fetal disorders** Fetal growth restriction Fetal hypokinesia Fetal heart rate abnormal Tachycardia fetal Amniorrhea	**166 (2.58)** 40 (0.62) 32 (0.50) 16 (0.25) 9 (0.14) 4 (0.06)	**99 (2.38)** 12 (0.29) 16 (0.38) 14 (0.34) 2 (0.05) 6 (0.14)	**14 (0.52)** - - 1 (0.04) 4 (0.15) 1 (0.04)	**7 (2.77)** - 3 (1.19) 1 (0.40) - -	**2 (1.65)** - 1 (0.83) 1 (0.83) - -	**288 (2.11)** 52 (0.38) 52 (0.38) 33 (0.24) 15 (0.11) 11 (0.08)
**Congenital, familial, and genetic disorders** Congenital anomaly Multiple congenital abnormalities Ventricular septal defect Heart disease congenital Atrial septal defect	**103 (1.60)** 12 (0.19) 3 (0.05) 3 (0.05) 6 (0.09) 4 (0.06)	**36 (0.86)** - 2 (0.05) 2 (0.05) - 2 (0.05)	**14 (0.52)** 1 (0.04) 1 (0.04) 1 (0.04) - -	**1 (0.40)** - - - - -	**2 (1.65)** - - - - -	**156 (1.14)** 13 (0.10) 6 (0.04) 6 (0.04) 6 (0.04) 6 (0.04)
**Neonatal disorders** Premature baby Low birth weight baby Death neonatal Jaundice neonatal Hypoglycemia neonatal	**97 (1.51)** 56 (0.87) 2 (0.03) 4 (0.06) 4 (0.06) 3 (0.05)	**32 (0.77)** 12 (0.29) 3 (0.07) 2 (0.05) 1 (0.02) 1 (0.02)	**11 (0.41)** 5 (0.19) 2 (0.07) 1 (0.04) - -	- - - - - -	- - - - - -	**140 (1.02)** 73 (0.53) 7 (0.05) 7 (0.05) 5 (0.04) 4 (0.03)
**Neonatal other AEFI** Cerebrovascular accident Pyrexia Cerebral infarction Death Anal fistula	**43 (0.67)** - 2 (0.03) 2 (0.03) 2 (0.03) 1 (0.02)	**8 (0.19)** - - - - -	**3 (0.11)** 2 (0.07) - - - -	**2 (0.79)** 1 (0.40) - - - -	**6 (4.96)** - - - - -	**62 (0.45)** 3 (0.02) 2 (0.01) 2 (0.01) 2 (0.01) 1 (0.01)
**Fetal other AEFI** Cerebral ventricle dilatation Ureteric dilatation Myocarditis Pericarditis Brain hypoxia	**13 (0.20)** 2 (0.03) 2 (0.03) 1 (0.02) 1 (0.02) 1 (0.02)	- - - - - -	- - - - - -	- - - - - -	- - - - - -	**13 (0.10)** 2 (0.01) 2 (0.01) 1 (0.01) 1 (0.01) 1 (0.01)
**PTs not indicating AEFI** Maternal exposure during pregnancy Exposure during pregnancy Maternal exposure timing unspecified Fetal exposure during pregnancy Inappropriate schedule of product administration	**1,873 (29.13)** 1170 (18.20) 239 (3.72) 105 (1.63) 45 (0.70) 83 (1.29)	**1,026 (24.63)** 547 (13.13) 364 (8.74) 2 (0.05) 13 (0.31) 8 (0.19)	**667 (24.79)** 379 (14.08) 158 (5.87) 2 (0.07) 40 (1.49) 2 (0.07)	**72 (28.46)** 9 (3.56) 35 (13.83) - 2 (0.79) 1 (0.40)	**39 (32.23)** 13 (10.74) 4 (3.31) - 3 (2.48) 1 (0.83)	**3,677 (26.92)** 2118 (15.51) 800 (5.86) 109 (0.80) 103 (0.75) 95 (0.70)

§ The first 5 mostly reported Preferred Terms were listed; the other Preferred Terms are reported in electronic **Supplementary Table S1**.

* Mix vaccination was represented by the following combinations: Oxford-AstraZeneca/Pfizer-BioNTech, Moderna/Pfizer-BioNTech, and Moderna/Oxford-AstraZeneca.

Data are expressed as n (%).

AEFI, adverse event following immunization; mRNA, messenger RNA; PTs, preferred terms.Bold values stand for the total number of each single groups.

Regarding non-pregnancy-specific adverse events, headache, pyrexia, fatigue, myalgia, and pain in extremity were the five most frequent local and systemic reactions in women exposed to COVID-19 vaccines during pregnancy; the 75.3% (n = 5,676) was caused by mRNA vaccines.

Among pregnancy-, neonatal-, or fetal-specific adverse events, 1,214 PTs (8.89%) belonged to the Event Group “Termination of pregnancy and risk of abortion,” 575 (4.21%) to “Pregnancy, labor, and delivery complications and risk factors (excluding abortions and stillbirth),” 288 (2.11%) to “Fetal disorders,” 156 (1.14%) to “Congenital, familial, and genetic disorders,” 140 (1.02%) to “Neonatal disorders,” 62 (0.45%) to “Neonatal other AEFI,” and 13 (0.10%) to “Fetal other AEFI.” The five most commonly reported PTs for the Event Group “Termination of pregnancy and risk of abortion” were as follows: abortion spontaneous (n = 952; 6.97%), fetal death (n = 89; 0.65%), abortion missed (n = 55; 0.40%), stillbirth (n = 28; 0.20%), and abortion (n = 27; 0.20%). The five most commonly reported PTs for the Event Group “Pregnancy, labor, and delivery complications and risk factors (excluding abortions and stillbirth)” were the following: hemorrhage in pregnancy (n = 69; 0.51%), uterine contractions during pregnancy (n = 58; 0.42%), premature labor (n = 56; 0.41%), premature delivery (n = 41; 0.30%), and preeclampsia (n = 28; 0.20%). Among “Fetal disorders,” the first five PTs were as follows: fetal growth restriction (n = 52; 0.38%), fetal hypokinesia (n = 52; 0.38%), fetal heart rate abnormal (n = 33; 0.24%), tachycardia fetal (n = 15; 0.11%), and amniorrhea (n = 11; 0.08%). Details on all PTs related to each COVID-19 vaccine were listed in **Supplementary Table S1**.

Overall, most of the unique ICSR-PT combinations were classified as serious (9,185; 67.24%). Specifically, the seriousness criteria were as follows: other medically important conditions (5,521; 40.42%), caused or prolonged hospitalization (1,734; 12.69%), congenital anomaly (648; 4.74%), life threatening (548; 4.01%), disabling (451; 3.30%), and results in death (283; 2.07%). In the Janssen vaccine group, no adverse event resulted in death or was disabling, but the proportion of events related to life threatening (42.69%) was higher than that of other COVID-19 vaccines (2.86% for Pfizer-BioNTech, 3.65% for Moderna, 3.64% for Oxford-AstraZeneca, and 4.96% for mix vaccination). Overall, the outcome was favorable for most events related to all COVID-19 vaccines (5,434; 39.78%) as compared with negative outcomes (2,884; 21.11%). However, a high percentage of unknown outcomes was reported (45.93% for Pfizer-BioNTech, 25.21% for Moderna, 43.74% for Oxford-AstraZeneca, 37.55% for Janssen, and 54.55% for mix vaccination). When outcomes were specified, most of them were favorable for Pfizer-BioNTech vaccine (33.3% *vs*. 20.77%), Moderna vaccine (52.53% *vs*. 22.26%), Oxford-AstraZeneca vaccine (27.83% *vs*. 19.44%), and Janssen vaccine (34.79% *vs*. 27.67%), while there was a similar proportion between favorable and unfavorable outcomes for mix vaccination (23.96% *vs*. 21.49%). Seriousness and outcome criteria for each COVID-19 vaccine are presented in **Table 3**.

**Table 3:** Seriousness and outcome of AEFI related to maternal exposure during pregnancy to COVID-19 vaccines reported in EudraVigilance from 1 January 2021 to 31 December 2021.

Variable	Level	mRNA vaccines*§*		Viral vector-based vaccines*§*		Mix vaccination*§ (n=121)	*Total§ *(n=13,659)
		Pfizer(n=6,429)	Moderna(n=4,165)	AstraZeneca(n=2,691)	Janssen(n=253)		
**Seriousness**	*Caused/Prolonged Hospitalization (%)*	554 (8.62)	758 (18.20)	354 (13.15)	42 (16.60)	26 (21.49)	1,734 (12.69)
	*Other Medically Important Condition (%)*	3,043 (47.33)	1,310 (31.45)	1,050 (39.02)	68 (26.88)	50 (41.32)	5,521 (40.42)
	*Life Threatening (%)*	184 (2.86)	152 (3.65)	98 (3.64)	108 (42.69)	6 (4.96)	548 (4.01)
	*Results in Death (%)*	203 (3.16)	10 (0.24)	68 (2.53)	–	2 (1.65)	283 (2.07)
	*Disabling (%)*	177 (2.75)	98 (2.35)	176 (6.54)	–	–	451 (3.30)
	*Congenital Anomaly (%)*	170 (2.64)	247 (5.93)	215 (7.99)	3 (1.19)	13 (10.74)	648 (4.74)
	*Not Serious (%)*	2,098 (32.63)	1,590 (38.18)	730 (27.13)	32 (12.65)	24 (19.83)	4,474 (32.75)
**Outcome**	*Recovered/Resolved (%)*	1,392 (21.65)	1,931 (46.36)	706 (26.24)	79 (31.23)	2 (9.92)	4,120 (30.16)
	*Recovering/Resolving (%)*	749 (11.65)	257 (6.17)	285 (10.59)	9 (3.56)	14 (11.57)	1,314 (9.62)
	*Not Recovered/Not Resolved (%)*	1,038 (16.15)	898 (21.56)	394 (14.64)	70 (27.67)	19 (15.70)	2,419 (17.71)
	*Fatal (%)*	203 (3.16)	10 (0.24)	68 (2.53)	–	2 (1.65)	283 (2.07)
	*Recovered/Resolved with Sequelae (%)*	94 (1.46)	19 (0.46)	61 (2.27)	–	8 (6.61)	182 (1.33)
	*Unknown (%)*	2,953 (45.93)	1,050 (25.21)	1,177 (43.74)	95 (37.55)	66 (54.55)	5,341 (39.10)

*Mix vaccination was represented by the following combinations: Oxford-AstraZeneca/Pfizer-BioNTech, Moderna/Pfizer-BioNTech, and Moderna/Oxford-AstraZeneca.

§ Total number of unique ICSR-PT combinations (superior to the number of ICSRs because an ICSR may be assigned one or more MedDRA PTs).

Data are expressed as n (%).

AEFI, adverse event following immunization; mRNA, messenger RNA.

### Fatal cases

A total of 283/13,659 (2.07%) events were classified as fatal (according to what was declared by the reporter) corresponding to 111/3,252 ICSRs. Moreover, 77/3,252 cases were reclassified as fatal. On a total of 188 fatal cases (5.8%), 17 were clearly associated with the mother, while 171 were associated with the fetus/newborn.

In the fatal ICSRs related to pregnant women, thrombotic events were reported in six cases (including 5 thrombosis with thrombocytopenia syndrome and 1 thrombosis not specified), cardiovascular events in four cases (including 1 cardiorespiratory arrest, 1 cardiac disorder, 1 hemorrhagic stroke, and 1 hypertension), suspected COVID-19 in three cases, respiratory distress in two cases, hemorrhagic event in one case, and event not specified in one case.

Among the ICSRs related to fetuses/newborns, 144 fatal cases occurred in fetal age and 27 in neonatal age. In the fatal cases related to the fetus, there were 49 fetal deaths not specified or without other relevant information, followed by 26 congenital malformations [7 congenital anomaly or fetal malformation not specified, 3 multiple congenital abnormalities, 3 hydrocephalus (1 with spina bifida), 2 congenital central nervous system anomalies, 2 cardiac malformations (1 hypoplastic left heart syndrome and atrial septal defect and 1 heart disease congenital), 1 limb malformation, 1 congenital absences of cranial vault, 1 macrocephaly/PTEN gene mutation, 1 neural tube defect, 1 pulmonary malformation, 1 renal aplasia, 1 syndactyly, 1 trisomy 18, and 1 trisomy 21]. In the remaining cases, there were 19 stillbirths without other information, 14 multiple events (8 fetal growth disturbances and cardiac disorders, 2 fetal hypokinesia and cardiac disorders, 1 cardiac disorder and hydrops fetalis, 1 fetal hypokinesia and umbilical cord abnormality, 1 fetal hypokinesia and cardiovascular disorder, and 1 fetal growth restriction and amniotic fluid disturbance), 13 fetal growth disturbances, 9 fetal hypokinesia, 7 cardiac disorders, 3 amniotic fluid and placental disturbances, 2 infections, 1 hemorrhagic events, and 1 hydrops fetalis.

Neonatal safety reports were characterized by 10 premature birth conditions (2 also with placental disorder and 2 with premature separation of placenta), 7 congenital malformations (2 heart disease congenital, 2 limb malformation, 2 multiple anomalies, and 1 hydrocephalus), 4 respiratory disorders (1 with meconium aspiration syndrome), 3 neonatal deaths without other reported events, 1 sepsis, 1 generalized edema, and 1 complex case in which the neonate presented hydranencephaly, neonatal necrotizing enterocolitis, neonatal seizure, and perinatal stroke. The distribution of fatal ICSRs related to mother, fetus, and infant by COVID-19 vaccines is reported in **Tables 4A–C**.

**Table 4:** Distribution of fatal cases of pregnant women (a), fetuses (b), and neonates (c) for each COVID-19 vaccine.

The most clinically relevant events reported in the ICSRs	mRNA vaccines		Viral Vector-Based vaccines		Mix vaccination* (n=21)	*Total*(n=3,252)
	Pfizer (n=1,645)	Moderna (n=967)	AstraZeneca (n=573)	Janssen (n=46)		
A						
Thrombotic events	1	–	5	–	–	6
Cardiovascular events	2	1	1	–	–	4
COVID-19	3	–	–	–	–	3
Respiratory events	1	–	1	–	–	2
Hemorrhagic events	–	–	–	–	1	1
Not specified events	–	–	1	–	–	1
***Total***	***7***	***1***	***8***	***-***	***1***	***17***
B						
Fetal death without other relevant information	21	25	2	–	1	49
Congenital malformations	24	1	1	–	–	26
Stillbirths without other relevant information	4	13	1	1	–	19
Multiple events	10	3	1	–	–	14
Fetal growth disturbances	11	1	1	–	–	13
Fetal hypokinesia	8	1	–	–	–	9
Cardiac disorders	7	–	–	–	–	7
Amniotic fluid and placental disturbances	3	–	–	–	–	3
Infection	1	–	1	–	–	2
Hemorrhagic events	1	–	–	–	–	1
Hydrops fetalis	–	1	–	–	–	1
***Total***	***90***	***45***	***7***	***1***	***1***	***144***
C						
Premature birth conditions	6	3	1	–	–	10
Congenital malformations	6	–	1	–	–	7
Respiratory disorders	4	–	–	–	–	4
Neonatal death without other relevant information	1	2	–	–	–	3
Infection	1	–	–	–	–	1
Generalized oedema	1	–	–	–	–	1
Multiple events	1	–	–	–	–	1
***Total***	***20***	***5***	***2***	***-***	***-***	***27***

*Mix vaccination was represented by the following combinations: Oxford-AstraZeneca/Pfizer-BioNTech, Moderna/Pfizer-BioNTech, and Moderna/Oxford-AstraZenecaI.ICSRs, Individual Case Safety Reports; mRNA, mesenger RNA.

### Reporting odds ratio (ROR) for spontaneous abortion

**Figure 2:**
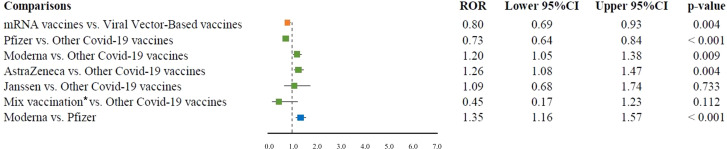
ROR of spontaneous abortion for the comparisons between COVID-19 vaccines. ROR, reporting odds ratio; CI, confidence interval; mRNA, messenger RNA. *Mix vaccination was represented by the following combinations: Oxford-AstraZeneca/Pfizer-BioNTech, Moderna/Pfizer-BioNTech, and Moderna/Oxford-AstraZeneca.

Among the pregnancy-related Event Groups, spontaneous abortion was the only PT reported for all COVID-19 vaccines and in more than three cases. mRNA vaccines had a lower reporting probability of spontaneous abortion than viral vector-based vaccines (ROR 0.80, 95% CI 0.69–0.93). In the comparison with the other COVID-19 vaccines, Moderna and Oxford-AstraZeneca vaccines had a higher reporting probability of spontaneous abortion (ROR 1.2, 95% CI 1.05–1.38, P = 0.009; and ROR 1.26, 95% CI 1.08–1.47, respectively). A lower reporting probability of spontaneous abortion was found for Pfizer-BioNTech vaccine compared with the other COVID-19 vaccines (ROR 0.73, 95% CI 0.64–0.84). Finally, no difference between Janssen vaccine or mix vaccinations compared to all the other COVID-19 vaccines was observed for spontaneous abortion (ROR 1.02, 95% CI 0.91–1.14; and ROR 1.12, 95% CI 0.94–1.34, respectively). Among mRNA vaccines, Moderna had a higher ROR than Pfizer-BioNTech (ROR 1.35, 95% CI 1.16–1.57) (**Figure 2**).

## Discussion

In this study, we investigated the safety profile of COVID-19 vaccines received by women during pregnancy in terms of maternal and fetal/neonatal AEFI by analyzing data from the EV database. Most ICSRs referred to mRNA vaccines probably because they were associated with a higher use than viral vector-based vaccines due to the EMA’s recommendation and evidence indicating the absence of pregnancy complications for expectant mothers and their babies with these vaccines.

Our study showed a high rate of serious ICSRs (87.82%), which could be related to several factors, such as the subpopulation represented by pregnant women, who are considered as a vulnerable or special population (15). However, although most cases were classified as serious, they were also associated with a favorable outcome. Additionally, it is interesting to discuss the main source of ICSRs. In fact, 58.24% of ICSRs were reported by non-HCPs. Generally, HCPs seem to be more prone to report serious AEFI/ADRs that result in hospitalization, are life threatening, or result in death (16–19). The higher involvement of these “*lay*” reporters reflects the great interest and concern on the use of these new COVID-19 vaccines in this special subpopulation.

The vaccine was the only suspect reported in about 97% of cases. This could highlight the tendency of avoiding pharmacological treatments, unless strictly necessary, by the gynecologists and the pregnant women or that the attention at the time was mainly focused on COVID-19 vaccination. From our results, more cases of AEFI occurred in pregnant women than in fetuses/newborns. We found that most events were not related to a pregnancy outcome (55.16%) but were mainly flu-like symptoms similar to those observed in the general population exposed to COVID-19 vaccines (20). Accordingly, Shimabukuro et al. (3) reported that in the United States, more than 35,691 pregnant women have been vaccinated with mRNA COVID-19 vaccines and a total of about 29,255 reports were received concerning local and systemic reactions. Additionally, a recent study found a lower rate of significant health events in pregnant women than non-pregnant individuals after COVID-19 vaccination (21).

Spontaneous abortion was the most reported AEFI (7%) in line with other published findings (22, 23). In fact, abortion was the most common reported AEFI also in other large pharmacovigilance databases, such as VAERS (3). This high reporting is similar to what was observed during the influenza A (H1N1) pandemic after the introduction of the related influenza vaccine in pregnant women (3, 24). Therefore, the high reporting of abortion may be driven by the media attention generated by the rapid development and introduction of COVID-19 vaccines that alarmed the scientific community and the general public. Generally, early pregnancy loss can have several causes; in particular, fetal chromosomal abnormalities seem to recover 50% of cases. Among other reasons, advanced maternal age and previous early pregnancy loss are common risk factors (23, 25). Other risk factors are alcohol and narcotic abuse or the presence of chronic diseases that can precipitate spontaneous abortion, such as diabetes, celiac disease, or autoimmune diseases (25). Unfortunately, we could not assess cases for the presence of risk factors due to the limited information available. Based on our pharmacovigilance dataset, we have performed a disproportionality analysis to compare the reporting probability of spontaneous abortion among COVID-19 vaccines. We found that Moderna and Oxford-AstraZeneca vaccines had about 20% higher reporting probability of spontaneous abortion than all other COVID-19 vaccines, while Pfizer-BioNTech vaccine had 27% lower reporting probability than all other COVID-19 vaccines. Accordingly, we found similar results for the direct comparison between grouped vaccine classes and within the vaccine class of mRNA vaccines. Previously, a case-control study on data from Norwegian registries on first-trimester pregnancies and COVID-19 vaccination evaluated the odds ratio of miscarriage before 14 weeks of gestation compared to unvaccinated pregnant women. The adjusted odds ratios were 0.91 (95% CI 0.75–1.10) for vaccination in the previous 3 weeks and 0.81 (0.69–0.95) for vaccination in the previous 5 weeks (26). A retrospective study analyzed data from the Centers for Disease Control and Prevention (CDC) v-safe mRNA COVID-19 vaccine pregnancy registry to determine the cumulative risk of spontaneous abortion from 6 to less than 20 weeks of gestation. This study found among 2,022 participants with ongoing pregnancies, a total of 165 spontaneous abortion (27). Moreover, the cumulative risk of spontaneous abortion was 14.1% (95% CI 12.1–16.1) in the primary analysis and it was in line with the expected risk range of two historical cohorts (27–29). A case–control surveillance study of mRNA COVID-19 vaccination during pregnancy and spontaneous abortion assessed the odds ratio of receiving the vaccine in the 28 days prior to spontaneous abortion compared with that in the 28 days prior to index dates for ongoing pregnancies. This study did not find increased odds of exposure to a COVID-19 vaccine in the prior 28 days compared with ongoing pregnancy (adjusted odds ratio 1.02; 95% CI 0.96–1.08) (30). All of these studies focused on the evaluation of mRNA COVID-19 vaccines probably because they were mostly used in this subpopulation, also in accordance with regulatory recommendation (31). Our results also showed that most cases of spontaneous abortion (74.2%) reported an mRNA COVID-19 vaccine. This highlights the paucity of data on viral vector COVID-19 vaccines and that further information is needed to complete the evaluation of pregnancy outcomes and to allow definitive recommendations from regulatory agencies.

Among congenital-, neonatal-, or fetal-specific adverse events, our findings did not indicate any safety concern among COVID-19 vaccines. This result is comfortable also because to date the regulatory agencies [e.g., EMA, US Food and Drug Administration (FDA)] did not detect from observational studies and pharmacovigilance databases any signal of alarm related to maternal exposure during pregnancy to COVID-19 vaccination both in mother and in unborn/newborn.

In our study, we found low reporting rates of congenital abnormalities (1.14%) and stillbirth (0.20%). Accordingly, a cohort study found that pregnancy and neonatal outcomes in pregnant women who have received at least one dose of COVID-19 vaccine were similar to a propensity score-matched cohort of pregnant women who did not receive the vaccine. A total of 133 women had similar rates of adverse pregnancy outcomes, of which stillbirth (0.0% *vs*. 0.3%) and fetal abnormalities (2.2% *vs*. 2.7%) (32). Continuous monitoring is needed to further assess these outcomes associated with maternal exposure to COVID-19 vaccines. Meanwhile, our results can be added to the available evidence to facilitate informed decision-making about vaccination in pregnant women. Moreover, considering the risks associated with the onset of COVID-19 for the mother or fetus, COVID-19 vaccination is relevant and warranted in this subpopulation.

In our study, 5.8% of ICSRs reported a fatal outcome, of which 0.5% was related to the mother, lower than that observed in pregnant women with COVID-19 (1.6%) (33). Regardless of COVID-19, using as data source the National Center for Health Statistics and the CDC’s WONDER database, Howard et al. (34) found that 9,532 pregnant women died from 2015 to 2019. The pregnancy-related mortality rate was 27.5 per 100,000 live births in 2019 (34). According to the Pregnancy Mortality Surveillance System (PMSS)-CDC, the number of reported pregnancy-related deaths (defined as the death of a woman while pregnant) in the United States was 17.3 deaths per 100,000 live births (in 2017), and the main causes were other cardiovascular conditions (15.5%), infection or sepsis (12.7%), cardiomyopathy (11.5%), hemorrhage (10.7%), thrombotic pulmonary or other embolism (9.6%), cerebrovascular accidents (8.2%), hypertensive disorders of pregnancy (6.6%), amniotic fluid embolism (5.5%), anesthesia complications (0.4%), other non-cardiovascular medical conditions (12.5%), and unknown cause (6.7%) (35). Despite rarity, thrombotic events after viral vector-based COVID-19 vaccines have initially alarmed the scientific and general community. We identified six cases of thrombotic events, of which five were related to Oxford-AstraZeneca and one to Pfizer-BioNTech. No case was observed for Moderna and Janssen vaccines. Instead, in the United States, cases have been previously reported with the Janssen vaccine, leading the FDA to release a safety warning on this potential risk (36).

Although a pharmacovigilance study cannot detect the exact causes of death (if not openly declared) because of its data source and applied methodology, the most clinically relevant events related to maternal deaths and reported in our ICSRs were in line with these data.

Although the percentage of fatal outcomes in fetal age was high (4.4%), these cases were very heterogeneous and a relevant part of them (49/171 = 28.6%) was characterized by fetal death without other information. Therefore, it is not possible to well define if the codifying “fetal death” represented an early pregnancy loss (abortion) or an advanced one for the reporter. Additionally, the fetal and neonatal congenital malformations represented a relevant part in these fatal cases (33/171 = 19.3%). According to the estimates of the European network of population-based registries for the epidemiological surveillance of congenital anomalies (EUROCAT), congenital anomalies are a leading cause of fetal death, infant mortality, and morbidity in childhood worldwide (37). The EUROCAT reported that each year in the European Union, approximately 125,000 fetuses and infants of 5.0 million births (2.5%) have a congenital anomaly (37). Another study reported that the incidence of congenital malformations among 5,739 products of conception (weighing over 500 g) was 7.5%; the rate was 7.0% among infants born alive and surviving the neonatal period, 13.6% among antepartum deaths, 23.3% among intrapartum deaths, and 29.6% among neonatal deaths (38).

### Strengths and limitations

To the best of our knowledge, the present study is the first based on data from the European spontaneous reporting system. It has several strengths. First and foremost, the EV is one of the largest databases of spontaneous reports of ADR and AEFI and, as shown also by other authors, this database offers the chance to analyze very large numbers of adverse events across national boundaries (39). Moreover, it allows us to take a picture of the actual tolerability profile of widely used vaccines during the pandemic. Lastly, this study focused on a special population that is generally excluded from clinical trials and limitedly investigated in observational studies, thus representing a valuable source of real-world data.

Our study also has expected limitations related to the passive surveillance (such as underreporting and poor quality of information). Among them, the poor quality of information limits our analysis from considering risk factors for pregnancy outcomes, the time between the vaccination during pregnancy and the onset of AEFI (relevant mostly for congenital anomalies and neonatal events), and the number of vaccine dose related to the AEFI. Furthermore, there are no available data on the number of vaccinated pregnant women in Europe, which hinders computation of reporting rates from EV data. Another limitation of our study is related to the analyzed special population because we often observed a common mistake in filling out the reporting form. Generally, the reporter finds it difficult to establish who should be the patient who is the subject of the report (e.g., in the case of abortion if the patient is the mother or the fetus).

Additionally, our study carries selection biases related to the identification of the pregnancy status, which cannot be automatically selected from EV but needs manual keyword selection strategies. Therefore, there is a chance that not all pregnancy cases reported in EV were included. Finally, an important consideration that should be stated for AEFI/ADR retrieved from spontaneous reporting systems, with greater attention for those fatal, is that we cannot be sure of their causal relationship with a vaccine or a drug. Regarding this, it is important to underline that in the pharmacovigilance field, spontaneous reporting is driven by the suspect and not the certainty of an AEFI/ADR associated with an administered vaccine or drug (40).

### Conclusions

Using data from the EV, we carried out a descriptive analysis of ICSRs in women exposed to COVID-19 vaccines during pregnancy. Our results demonstrated that 0.25% (3,252/1,315,315) of ICSRs reporting COVID-19 vaccines as suspected vaccines described the occurrence of AEFI in pregnant women and fetuses/newborns. Considering that the risks following SARS-CoV-2 infection and especially the risk of mortality are much greater than those associated with vaccinations in pregnant women, this low reporting frequency highlights the multiple benefits of COVID-19 vaccination programs also in pregnant women.

In conclusion, according to the actual positive opinion of regulatory agencies for the use of COVID-19 vaccines in pregnancy, we did not observe any strong insight of unknown adverse events associated with COVID-19 vaccination in pregnancy. However, we analyzed data related to 1 year (the first) of vaccination program. Therefore, our experience also highlights the need for continuing to monitor the safety profile of COVID-19 vaccines and to identify long-term adverse events following immunization.

## Data availability statement

The original contributions presented in the study are included in the article/**Supplementary Material**. Further inquiries can be directed to the corresponding author.

## Ethics statement

Ethical review and approval was not required for the study on human participants in accordance with the local legislation and institutional requirements. Written informed consent from the patients/participants or patients/participants’ legal guardian/next of kin was not required to participate in this study in accordance with the national legislation and the institutional requirements.

## Author contributions

AM, GM, FF, MG, AZ, CP, FR, AC and LS. Drafting the work and revising it for important intellectual content; AM, GM, FF, MG, AZ, CP, FR, AC and LS. Substantial contributions to the acquisition, analysis, or interpretation of data for the work; AM, GM, FF, MG, AZ, CP, FR, AC and LS. Final approval of the version to be published; AM, GM, FF, MG, AZ, CP, FR, AC and LS: Agreement to be accountable for all aspects of the work in ensuring that questions related to the accuracy or integrity of any part of the work are appropriately investigated and resolved. FR, AC and LS: Developed the concept. AM, GM, FF and LS: Wrote the paper. All authors contributed to the article and approved the submitted version.

## Conflict of interest

The authors declare that the research was conducted in the absence of any commercial or financial relationships that could be construed as a potential conflict of interest.

## Publisher’s note

All claims expressed in this article are solely those of the authors and do not necessarily represent those of their affiliated organizations, or those of the publisher, the editors and the reviewers. Any product that may be evaluated in this article, or claim that may be made by its manufacturer, is not guaranteed or endorsed by the publisher.

## References

[1] European Medicines Agency (EMA) . COVID-19 vaccines (2022). Available at: https://www.ema.europa.eu/en/human-regulatory/overview/public-health-threats/coronavirus-disease-covid-19/treatments-vaccines/covid-19-vaccines (Accessed April 13, 2022).

[2] Kadali RAK Janagama R Peruru SR Racherla S Tirumala R Madathala RR . Adverse effects of COVID-19 messenger RNA vaccines among pregnant women: a cross-sectional study on healthcare workers with detailed self-reported symptoms. Am J Obstet Gynecol (2021) 225(4):458–60. doi: 10.1016/j.ajog.2021.06.007 PMC8189739 34118200

[3] Shimabukuro TT Kim SY Myers TR Moro PL Oduyebo T Panagiotakopoulos L . Preliminary findings of mRNA covid-19 vaccine safety in pregnant persons. N Engl J Med (2021) 384(24):2273–82. doi: 10.1056/NEJMoa2104983 PMC8117969 33882218

[4] Fell DB Dhinsa T Alton GD Török E Dimanlig-Cruz S Regan AK . Association of COVID-19 vaccination in pregnancy with adverse peripartum outcomes. JAMA (2022) 327(15):1478–87. doi: 10.1001/jama.2022.4255 PMC8949767 35323842

[5] Rottenstreich M Sela HY Rotem R Kadish E Wiener-Well Y Grisaru-Granovsky S . Covid-19 vaccination during the third trimester of pregnancy: Rate of vaccination and maternal and neonatal outcomes, a multicentre retrospective cohort study. BJOG (2022) 129(2):248–55. doi: 10.1111/1471-0528.16941 PMC8652528 34554630

[6] Larocca RA Mendes EA Abbink P Peterson RL Martinot AJ Iampietro MJ . Adenovirus vector-based vaccines confer maternal-fetal protection against zika virus challenge in pregnant IFN-αβR-/- mice. Cell Host Microbe (2019) 26(5):591–600.e4. doi: 10.1016/j.chom.2019.10.001 31668877 PMC6863051

[7] Dashraath P Nielsen-Saines K Madhi SA Baud D . COVID-19 vaccines and neglected pregnancy. Lancet (2020) 396(10252):e22. doi: 10.1016/S0140-6736(20)31822-5 32861313 PMC7723327

[8] Rouse CE Eckert LO Babarinsa I Fay E Gupta M Harrison MS . Spontaneous abortion and ectopic pregnancy: Case definition & guidelines for data collection, analysis, and presentation of maternal immunization safety data. Vaccine (2017) 35(48 Pt A):6563–74. doi: 10.1016/j.vaccine.2017.01.047 PMC5714431 29150062

[9] International Conference of Harmonization (ICH) . Introductory guide MedDRA version 24.1 (2021). Available at: https://admin.meddra.org/sites/default/files/guidance/file/000594_intguide_%2024_1.pdf (Accessed April 13, 2022).

[10] European Medicine Agency (EMA) . Important medical event terms list - MedDRA version 25.0 (2022). Available at: https://www.ema.europa.eu/en/documents/other/meddra-important-medical-event-terms-list-version-250_en.xlsx (Accessed August 22, 2022).

[11] Citu IM Citu C Gorun F Motoc A Gorun OM Burlea B . Determinants of COVID-19 vaccination hesitancy among Romanian pregnant women. Vaccines (2022) 10(2):275. doi: 10.3390/vaccines10020275 35214732 PMC8874778

[12] Sessa M Rafaniello C Scavone C Mascolo A di Mauro G Fucile A . Preventable statin adverse reactions and therapy discontinuation. What can we learn from the spontaneous reporting system? Expert Opin Drug Saf (2018) 17(5):457–65. doi: 10.1080/14740338.2018.1458837 29619841

[13] Ruggiero R Fraenza F Scavone C di Mauro G Piscitelli R Mascolo A . Immune checkpoint inhibitors and immune-related adverse drug reactions: Data from Italian pharmacovigilance database. Front Pharmacol (2020) 11:830. doi: 10.3389/FPHAR.2020.00830 32581796 PMC7295943

[14] Mascolo A Scavone C Ferrajolo C Rafaniello C Danesi R Del Re M . Immune checkpoint inhibitors and cardiotoxicity: An analysis of spontaneous reports in eudravigilance. Drug Saf (2021) 44(9):957–71. doi: 10.1007/s40264-021-01086-8 PMC8370948 34145536

[15] European Medicines Agency (EMA) . Guideline on good pharmacovigilance practices (GVP). product- or population-specific considerations III: Pregnant and breastfeeding women. EMA/653036/2019 DRAFT FOR PUBLIC CONSULTATION (2019). Available at: https://www.ema.europa.eu/en/documents/scientific-guideline/draft-guideline-good-pharmacovigilance-practices-product-population-specific-considerations-iii_en.pdf (Accessed June 8, 2022).

[16] Banovac M Candore G Slattery J Houÿez F Haerry D Genov G . Patient reporting in the EU: Analysis of EudraVigilance data. Drug Saf (2017) 40(7):629–45. doi: 10.1007/s40264-017-0534-1 28417320

[17] di Mauro G Zinzi A Scavone C Mascolo A Gaio M Sportiello L . PCSK9 inhibitors and neurocognitive adverse drug reactions: Analysis of individual case safety reports from the eudravigilance database. Drug Saf (2021) 44(3):337–49. doi: 10.1007/s40264-020-01021-3 PMC7892743 33351170

[18] Parretta E Rafaniello C Magro L Coggiola Pittoni A Sportiello L Ferrajolo C . Improvement of patient adverse drug reaction reporting through a community pharmacist-based intervention in the campania region of Italy. Expert Opin Drug Saf (2014) 13(Suppl 1):S21–9. doi: 10.1517/14740338.2014.939582 25171156

[19] Mascolo A Ruggiero R Sessa M Scavone C Sportiello L Rafaniello C . Preventable cases of oral anticoagulant-induced bleeding: Data from the spontaneous reporting system. Front Pharmacol (2019) 10:425. doi: 10.3389/fphar.2019.00425 31114497 PMC6503045

[20] Gringeri M Mosini G Battini V Cammarata G Guarnieri G Carnovale C . Preliminary evidence on the safety profile of BNT162b2 (Comirnaty): New insights from data analysis in EudraVigilance and adverse reaction reports from an Italian health facility. Hum Vaccin Immunother (2021) 17(9):2969–71. doi: 10.1080/21645515.2021.1917236 PMC8171003 34043934

[21] Sadarangani M Soe P Shulha HP Valiquette L Vanderkooi OG Kellner JD . Safety of COVID-19 vaccines in pregnancy: A Canadian national vaccine safety (CANVAS) network cohort study. Lancet Infect Dis (2022) S1473-3099(22):00426–1. doi: 10.1016/S1473-3099(22)00426-1 PMC9371587 35964614

[22] Trostle ME Limaye MA Avtushka V Lighter JL Penfield CA Roman AS . COVID-19 vaccination in pregnancy: Early experience from a single institution. Am J Obstet Gynecol MFM (2021) 3(6):100464. doi: 10.1016/j.ajogmf.2021.100464 34411758 PMC8366042

[23] American College of Obstetricians and Gynecologists’ Committee on Practice Bulletins—Gynecology . ACOG practice bulletin no. 200: early pregnancy loss. Obstet Gynecol (2018) 132(5):e197–207. doi: 10.1016/j.ajogmf.2021.100464 30157093

[24] Moro PL Broder K Zheteyeva Y Revzina N Tepper N Kissin D . Adverse events following administration to pregnant women of influenza a (H1N1) 2009 monovalent vaccine reported to the vaccine adverse event reporting system. Am J Obstet Gynecol (2011) 205(5):473. doi: 10.1016/j.ajog.2011.06.047 21861964 PMC6602056

[25] Nybo Andersen AM Wohlfahrt J Christens P Olsen J Melbye M . Maternal age and fetal loss: Population based register linkage study. BMJ (2000) 320(7251):1708–12. doi: 10.1136/bmj.320.7251.1708 PMC27416 10864550

[26] Magnus MC Gjessing HK Eide HN Wilcox AJ Fell DB Håberg SE . Covid-19 vaccination during pregnancy and first-trimester miscarriage. N Engl J Med (2021) 385(21):2008–10. doi: 10.1056/NEJMc2114466 PMC8552533 34670062

[27] Zauche LH Wallace B Smoots AN Olson CK Oduyebo T Kim SY . Receipt of mRNA covid-19 vaccines and risk of spontaneous abortion. N Engl J Med (2021) 385(16):1533–35. doi: 10.1056/NEJMc2113891 PMC8451181 34496196

[28] Mukherjee S Velez Edwards DR Baird DD Savitz DA Hartmann KE . Risk of miscarriage among black women and white women in a U. S Prospective Cohort Study Am J Epidemiol (2013) 177(11):1271–8. doi: 10.1093/aje/kws393 PMC3664339 23558353

[29] Goldhaber MK Fireman BH . The fetal life table revisited: Spontaneous abortion rates in three kaiser permanente cohorts. Epidemiology (1991) 2(1):33–9. doi: 10.1097/00001648-199101000-00006 2021664

[30] Kharbanda EO Haapala J DeSilva M Vazquez-Benitez G Vesco KK Naleway AL . Spontaneous abortion following COVID-19 vaccination during pregnancy. JAMA (2021) 326(16):1629–31. doi: 10.1001/jama.2021.15494 PMC8427483 34495304

[31] European Medicines Agency (EMA) . COVID-19: latest safety data provide reassurance about use of mRNA vaccines during pregnancy 2020. Available at: https://www.ema.europa.eu/en/news/covid-19-latest-safety-data-provide-reassurance-about-use-mrna-vaccines-during-pregnancy (Accessed June 8, 2022).

[32] Blakeway H Prasad S Kalafat E Heath PT Ladhani SN Le Doare K . COVID-19 vaccination during pregnancy: Coverage and safety. Am J Obstet Gynecol (2022) 226(2):236.e1–236.e14. doi: 10.1016/j.ajog.2021.08.007 34389291 PMC8352848

[33] Villar J Ariff S Gunier RB Thiruvengadam R Rauch S Kholin A . Maternal and neonatal morbidity and mortality among pregnant women with and without COVID-19 infection: The INTERCOVID multinational cohort study. JAMA Pediatr (2021) 175(8):817–26. doi: 10.1001/jamapediatrics.2021.1050 PMC8063132 33885740

[34] Howard JT Sparks CS Santos-Lozada AR Olowolaju SA Janak JC Howard KJ . Trends in mortality among pregnant and recently pregnant women in the US, 2015-2019. JAMA (2021) 326(16):1631–33. doi: 10.1001/jama.2021.13971 PMC8548946 34698794

[35] Centers for Disease Control and Prevention (CDC) . Reproductive health. pregnancy mortality surveillance system (2022). Available at: https://www.cdc.gov/reproductivehealth/maternal-mortality/pregnancy-mortality-surveillance-system.htm (Accessed June 8, 2022).

[36] Food and Drug Administration (FDA) . Fact sheet for healthcare providers administering vaccine (vaccination providers). Emergency use authorization (EUA) of the janssen covid-19 vaccine to prevent coronavirus disease 2019 (Covid-19). Available at: https://www.fda.gov/media/146304/download (Accessed August 22, 2022).

[37] Kinsner-Ovaskainen A Perraud A Lanzoni M Morris J Garne E . European Monitoring of congenital anomalies. JRC-EUROCAT report on statistical monitoring of congenital anomalies (2009-2018) (2021). Available at: https://eu-rd-platform.jrc.ec.europa.eu/system/files/public/EUROCAT-Statistical-Monitoring-Report-2021.pdf (Accessed June 8, 2022).

[38] Mcintosh R Merritt KK Richards MR Samuels MH Bellows MT . The incidence of congenital malformations: A study of 5,964 pregnancies. Pediatrics (1954) 14(5):505–22. doi: 10.1542/peds.14.5.505 13214966

[39] Blake KV Zaccaria C Domergue F La Mache E Saint-Raymond A Hidalgo-Simon A . Comparison between paediatric and adult suspected adverse drug reactions reported to the European medicines agency: Implications for pharmacovigilance. Paediatr Drugs (2014) 16(4):309–19. doi: 10.1007/s40272-014-0076-2 24898717

[40] European Medicines Agency (EMA) . Guideline on good pharmacovigilance practices (GVP) module VI – collection, management and submission of reports of suspected adverse reactions to medicinal products (Rev 2) 28 July 2017 EMA/873138/2011 rev 2* (2017). Available at: https://www.ema.europa.eu/en/documents/regulatory-procedural-guideline/guideline-good-pharmacovigilance-practices-gvp-module-vi-collection-management-submission-reports_en.pdf (Accessed June 8, 2022).

