# Supplementary material for: Maternal, fetal and neonatal outcomes among pregnant women receiving COVID-19 vaccination: The preg-co-vax study
Source: Front Immunol. 2022 Oct 3;13:965171. doi: 10.3389/fimmu.2022.965171 (PMC9574088; doi:10.3389/fimmu.2022.965171)
Supplement: Supplementary file 1 [file Table_1.docx]

**Supplementary table 1**. List of Preferred Terms (PTs) and their Standard MedDRA Queries (SMQs) related to Pfizer-BioNTech (A), Moderna (B), Oxford-AstraZeneca (C), Janssen vaccine (D), and Mix vaccination (E).

**A) Pfizer-BioNTech vaccine**

| **PT** | **SMQ** | **N** |
| --- | --- | --- |
| Abortion spontaneous | Termination of pregnancy and risk of abortion | 381 |
| Headache | Maternal other AEFI | 211 |
| Fatigue | Maternal other AEFI | 173 |
| Pyrexia | Maternal other AEFI | 126 |
| Pain in extremity | Maternal other AEFI | 110 |
| Nausea | Maternal other AEFI | 102 |
| Myalgia | Maternal other AEFI | 98 |
| Malaise | Maternal other AEFI | 83 |
| Vaccination site pain | Maternal other AEFI | 83 |
| Pain | Maternal other AEFI | 75 |
| Chills | Maternal other AEFI | 75 |
| Dizziness | Maternal other AEFI | 71 |
| Vaginal haemorrhage | Maternal other AEFI | 70 |
| Vomiting | Maternal other AEFI | 70 |
| Premature baby | Neonatal disorders | 56 |
| Dyspnoea | Maternal other AEFI | 51 |
| Arthralgia | Maternal other AEFI | 49 |
| Uterine contractions during pregnancy | Pregnancy, labour and delivery complications and risk factors (excl abortions and stillbirth) | 44 |
| Foetal death | Termination of pregnancy and risk of abortion | 41 |
| Lymphadenopathy | Maternal other AEFI | 40 |
| Foetal growth restriction | Foetal disorders | 40 |
| Abdominal pain | Maternal other AEFI | 37 |
| COVID-19 | Maternal other AEFI | 37 |
| Diarrhoea | Maternal other AEFI | 37 |
| Abortion missed | Termination of pregnancy and risk of abortion | 35 |
| Haemorrhage in pregnancy | Pregnancy, labour and delivery complications and risk factors (excl abortions and stillbirth) | 35 |
| Drug ineffective | Maternal other AEFI | 34 |
| Palpitations | Maternal other AEFI | 34 |
| Foetal hypokinesia | Foetal disorders | 32 |
| Asthenia | Maternal other AEFI | 30 |
| Chest pain | Maternal other AEFI | 29 |
| Haemorrhage | Maternal other AEFI | 29 |
| Cough | Maternal other AEFI | 28 |
| Heart rate increased | Maternal other AEFI | 27 |
| Influenza like illness | Maternal other AEFI | 24 |
| Premature labour | Pregnancy, labour and delivery complications and risk factors (excl abortions and stillbirth) | 24 |
| Pruritus | Maternal other AEFI | 23 |
| Oropharyngeal pain | Maternal other AEFI | 23 |
| Syncope | Maternal other AEFI | 22 |
| Tachycardia | Maternal other AEFI | 21 |
| Injection site pain | Maternal other AEFI | 21 |
| Hypoaesthesia | Maternal other AEFI | 20 |
| Back pain | Maternal other AEFI | 20 |
| Menstruation irregular | Maternal other AEFI | 20 |
| Hyperhidrosis | Maternal other AEFI | 19 |
| Illness | Maternal other AEFI | 19 |
| Feeling hot | Maternal other AEFI | 18 |
| Rash | Maternal other AEFI | 18 |
| Muscle spasms | Maternal other AEFI | 18 |
| Seizure | Maternal other AEFI | 17 |
| Vision blurred | Maternal other AEFI | 17 |
| Nasopharyngitis | Maternal other AEFI | 16 |
| Abdominal pain upper | Maternal other AEFI | 16 |
| Foetal heart rate abnormal | Foetal disorders | 16 |
| Peripheral swelling | Maternal other AEFI | 15 |
| Paraesthesia | Maternal other AEFI | 15 |
| Tremor | Maternal other AEFI | 15 |
| Feeling abnormal | Maternal other AEFI | 15 |
| Insomnia | Maternal other AEFI | 15 |
| Somnolence | Maternal other AEFI | 14 |
| Axillary pain | Maternal other AEFI | 14 |
| Heavy menstrual bleeding | Maternal other AEFI | 14 |
| Dysmenorrhoea | Maternal other AEFI | 13 |
| Swelling | Maternal other AEFI | 12 |
| Decreased appetite | Maternal other AEFI | 12 |
| Thrombosis | Maternal other AEFI | 12 |
| Neck pain | Maternal other AEFI | 12 |
| Migraine | Maternal other AEFI | 12 |
| Rhinorrhoea | Maternal other AEFI | 12 |
| Preterm premature rupture of membranes | Pregnancy, labour and delivery complications and risk factors (excl abortions and stillbirth) | 12 |
| Congenital anomaly | Congenital, familial and genetic disorders | 12 |
| Urticaria | Maternal other AEFI | 11 |
| Musculoskeletal stiffness | Maternal other AEFI | 11 |
| Lethargy | Maternal other AEFI | 11 |
| Menstruation delayed | Maternal other AEFI | 11 |
| Premature rupture of membranes | Pregnancy, labour and delivery complications and risk factors (excl abortions and stillbirth) | 11 |
| Premature delivery | Pregnancy, labour and delivery complications and risk factors (excl abortions and stillbirth) | 11 |
| Erythema | Maternal other AEFI | 10 |
| Blood pressure increased | Maternal other AEFI | 10 |
| Intermenstrual bleeding | Maternal other AEFI | 10 |
| Hot flush | Maternal other AEFI | 10 |
| Vaccination site erythema | Maternal other AEFI | 9 |
| Deep vein thrombosis | Maternal other AEFI | 9 |
| Sensitive skin | Maternal other AEFI | 9 |
| Chest discomfort | Maternal other AEFI | 9 |
| Vaccination site swelling | Maternal other AEFI | 9 |
| Body temperature increased | Maternal other AEFI | 9 |
| Tinnitus | Maternal other AEFI | 9 |
| Lymph node pain | Maternal other AEFI | 9 |
| Limb discomfort | Maternal other AEFI | 9 |
| Ectopic pregnancy | Pregnancy, labour and delivery complications and risk factors (excl abortions and stillbirth) | 9 |
| Tachycardia foetal | Foetal disorders | 9 |
| Pulmonary embolism | Maternal other AEFI | 8 |
| Cold sweat | Maternal other AEFI | 8 |
| Herpes zoster | Maternal other AEFI | 8 |
| Stillbirth | Termination of pregnancy and risk of abortion | 8 |
| Gestational diabetes | Pregnancy, labour and delivery complications and risk factors (excl abortions and stillbirth) | 8 |
| Respiratory distress | Maternal other AEFI | 7 |
| Bell's palsy | Maternal other AEFI | 7 |
| Ear pain | Maternal other AEFI | 7 |
| Menstrual disorder | Maternal other AEFI | 7 |
| Hypokinesia | Maternal other AEFI | 7 |
| Myocarditis | Maternal other AEFI | 7 |
| Injection site swelling | Maternal other AEFI | 7 |
| Influenza | Maternal other AEFI | 7 |
| Nasal congestion | Maternal other AEFI | 7 |
| Abortion induced | Termination of pregnancy and risk of abortion | 7 |
| Caesarean section | Pregnancy, labour and delivery complications and risk factors (excl abortions and stillbirth) | 7 |
| Premature separation of placenta | Pregnancy, labour and delivery complications and risk factors (excl abortions and stillbirth) | 7 |
| Foetal heart rate decreased | Foetal disorders | 7 |
| Vaccination site movement impairment | Maternal other AEFI | 6 |
| Gait disturbance | Maternal other AEFI | 6 |
| Facial paralysis | Maternal other AEFI | 6 |
| Vertigo | Maternal other AEFI | 6 |
| Abdominal discomfort | Maternal other AEFI | 6 |
| Hypotension | Maternal other AEFI | 6 |
| Urinary tract infection | Maternal other AEFI | 6 |
| Presyncope | Maternal other AEFI | 6 |
| Epistaxis | Maternal other AEFI | 6 |
| Hypoaesthesia oral | Maternal other AEFI | 6 |
| Confusional state | Maternal other AEFI | 6 |
| Visual impairment | Maternal other AEFI | 6 |
| Condition aggravated | Maternal other AEFI | 6 |
| Feeling cold | Maternal other AEFI | 6 |
| Placental disorder | Pregnancy, labour and delivery complications and risk factors (excl abortions and stillbirth) | 6 |
| Oligohydramnios | Pregnancy, labour and delivery complications and risk factors (excl abortions and stillbirth) | 6 |
| Pre-eclampsia | Pregnancy, labour and delivery complications and risk factors (excl abortions and stillbirth) | 6 |
| Foetal malformation | Foetal disorders | 6 |
| Foetal movement disorder | Foetal disorders | 6 |
| Heart disease congenital | Congenital, familial and genetic disorders | 6 |
| Neuralgia | Maternal other AEFI | 5 |
| Blood pressure decreased | Maternal other AEFI | 5 |
| Tenderness | Maternal other AEFI | 5 |
| Discomfort | Maternal other AEFI | 5 |
| Loss of consciousness | Maternal other AEFI | 5 |
| Dysgeusia | Maternal other AEFI | 5 |
| Hypertension | Maternal other AEFI | 5 |
| Anxiety | Maternal other AEFI | 5 |
| Burning sensation | Maternal other AEFI | 5 |
| Injection site erythema | Maternal other AEFI | 5 |
| Injection site haematoma | Maternal other AEFI | 5 |
| Hypersensitivity | Maternal other AEFI | 5 |
| Ageusia | Maternal other AEFI | 5 |
| Injection site inflammation | Maternal other AEFI | 5 |
| Abortion | Termination of pregnancy and risk of abortion | 5 |
| Anembryonic gestation | Termination of pregnancy and risk of abortion | 5 |
| Loss of personal independence in daily activities | Maternal other AEFI | 4 |
| Pelvic pain | Maternal other AEFI | 4 |
| Oedema | Maternal other AEFI | 4 |
| Uterine spasm | Maternal other AEFI | 4 |
| Vaginal discharge | Maternal other AEFI | 4 |
| Abdominal distension | Maternal other AEFI | 4 |
| Flushing | Maternal other AEFI | 4 |
| Streptococcal urinary tract infection | Maternal other AEFI | 4 |
| Abdominal pain lower | Maternal other AEFI | 4 |
| Uterine haemorrhage | Maternal other AEFI | 4 |
| Pericarditis | Maternal other AEFI | 4 |
| Tension headache | Maternal other AEFI | 4 |
| Sinusitis | Maternal other AEFI | 4 |
| Vaccination site warmth | Maternal other AEFI | 4 |
| Asthma | Maternal other AEFI | 4 |
| Sleep disorder | Maternal other AEFI | 4 |
| Anosmia | Maternal other AEFI | 4 |
| Postpartum haemorrhage | Pregnancy, labour and delivery complications and risk factors (excl abortions and stillbirth) | 4 |
| Hypertension | Pregnancy, labour and delivery complications and risk factors (excl abortions and stillbirth) | 4 |
| Subchorionic haematoma | Pregnancy, labour and delivery complications and risk factors (excl abortions and stillbirth) | 4 |
| Jaundice neonatal | Neonatal disorders | 4 |
| Death neonatal | Neonatal disorders | 4 |
| Foetal vascular malperfusion | Foetal disorders | 4 |
| Umbilical cord abnormality | Foetal disorders | 4 |
| Amniorrhoea | Foetal disorders | 4 |
| Foetal distress syndrome | Foetal disorders | 4 |
| Foetal cardiac arrest | Foetal disorders | 4 |
| Atrial septal defect | Congenital, familial and genetic disorders | 4 |
| Congenital central nervous system anomaly | Congenital, familial and genetic disorders | 4 |
| Bedridden | Maternal other AEFI | 3 |
| Eye swelling | Maternal other AEFI | 3 |
| Facial pain | Maternal other AEFI | 3 |
| Muscle twitching | Maternal other AEFI | 3 |
| Breast pain | Maternal other AEFI | 3 |
| Human chorionic gonadotropin decreased | Maternal other AEFI | 3 |
| Constipation | Maternal other AEFI | 3 |
| Pallor | Maternal other AEFI | 3 |
| Vaccination site rash | Maternal other AEFI | 3 |
| Rash pruritic | Maternal other AEFI | 3 |
| Cardiac flutter | Maternal other AEFI | 3 |
| Paraesthesia oral | Maternal other AEFI | 3 |
| Dizziness postural | Maternal other AEFI | 3 |
| Vaccination site bruising | Maternal other AEFI | 3 |
| Cellulitis | Maternal other AEFI | 3 |
| Inflammation | Maternal other AEFI | 3 |
| Axillary mass | Maternal other AEFI | 3 |
| Angina pectoris | Maternal other AEFI | 3 |
| Sinus tachycardia | Maternal other AEFI | 3 |
| Movement disorder | Maternal other AEFI | 3 |
| Vaccination site mass | Maternal other AEFI | 3 |
| Dysphagia | Maternal other AEFI | 3 |
| Thirst | Maternal other AEFI | 3 |
| Amenorrhoea | Maternal other AEFI | 3 |
| Muscular weakness | Maternal other AEFI | 3 |
| Dehydration | Maternal other AEFI | 3 |
| Haematoma | Maternal other AEFI | 3 |
| Disease recurrence | Maternal other AEFI | 3 |
| Hypersomnia | Maternal other AEFI | 3 |
| Morning sickness | Maternal other AEFI | 3 |
| Photophobia | Maternal other AEFI | 3 |
| Mouth ulceration | Maternal other AEFI | 3 |
| Myelitis transverse | Maternal other AEFI | 3 |
| Paralysis | Maternal other AEFI | 3 |
| Monoplegia | Maternal other AEFI | 3 |
| Gastrooesophageal reflux disease | Maternal other AEFI | 3 |
| Night sweats | Maternal other AEFI | 3 |
| Pharyngeal swelling | Maternal other AEFI | 3 |
| Abortion spontaneous complete | Termination of pregnancy and risk of abortion | 3 |
| Amniotic cavity infection | Pregnancy, labour and delivery complications and risk factors (excl abortions and stillbirth) | 3 |
| HELLP syndrome | Pregnancy, labour and delivery complications and risk factors (excl abortions and stillbirth) | 3 |
| Threatened labour | Pregnancy, labour and delivery complications and risk factors (excl abortions and stillbirth) | 3 |
| Placenta praevia haemorrhage | Pregnancy, labour and delivery complications and risk factors (excl abortions and stillbirth) | 3 |
| Complication of pregnancy | Pregnancy, labour and delivery complications and risk factors (excl abortions and stillbirth) | 3 |
| Hypoglycaemia neonatal | Neonatal disorders | 3 |
| Neonatal respiratory distress | Neonatal disorders | 3 |
| Bradycardia foetal | Foetal disorders | 3 |
| Foetal cardiac disorder | Foetal disorders | 3 |
| Foetal growth abnormality | Foetal disorders | 3 |
| Multiple congenital abnormalities | Congenital, familial and genetic disorders | 3 |
| Ventricular septal defect | Congenital, familial and genetic disorders | 3 |
| Trisomy 21 | Congenital, familial and genetic disorders | 3 |
| Anomalous pulmonary venous connection | Congenital, familial and genetic disorders | 3 |
| Heterotaxia | Congenital, familial and genetic disorders | 3 |
| Limb malformation | Congenital, familial and genetic disorders | 3 |
| Limb reduction defect | Congenital, familial and genetic disorders | 3 |
| Cerebral infarction | Neonatal other AEFI | 2 |
| Death | Neonatal other AEFI | 2 |
| Pyrexia | Neonatal other AEFI | 2 |
| Cerebral ventricle dilatation | Foetal other AEFI | 2 |
| Ureteric dilatation | Foetal other AEFI | 2 |
| Tachypnoea | Maternal other AEFI | 2 |
| Anaphylactic reaction | Maternal other AEFI | 2 |
| Genital herpes | Maternal other AEFI | 2 |
| Anaemia | Maternal other AEFI | 2 |
| Weight decreased | Maternal other AEFI | 2 |
| Infection | Maternal other AEFI | 2 |
| Deafness | Maternal other AEFI | 2 |
| Eye disorder | Maternal other AEFI | 2 |
| Hyperacusis | Maternal other AEFI | 2 |
| Neuritis | Maternal other AEFI | 2 |
| Cerebral venous sinus thrombosis | Maternal other AEFI | 2 |
| Labour pain | Maternal other AEFI | 2 |
| Peripheral coldness | Maternal other AEFI | 2 |
| Ovarian vein thrombosis | Maternal other AEFI | 2 |
| Panic disorder | Maternal other AEFI | 2 |
| Decreased embryo viability | Maternal other AEFI | 2 |
| Dysarthria | Maternal other AEFI | 2 |
| Dysstasia | Maternal other AEFI | 2 |
| Memory impairment | Maternal other AEFI | 2 |
| Induration | Maternal other AEFI | 2 |
| Nodule | Maternal other AEFI | 2 |
| Hypomenorrhoea | Maternal other AEFI | 2 |
| Mass | Maternal other AEFI | 2 |
| Eye pain | Maternal other AEFI | 2 |
| Speech disorder | Maternal other AEFI | 2 |
| Body temperature decreased | Maternal other AEFI | 2 |
| Disturbance in attention | Maternal other AEFI | 2 |
| Blister | Maternal other AEFI | 2 |
| Eczema | Maternal other AEFI | 2 |
| Cardiac discomfort | Maternal other AEFI | 2 |
| Cardiac infection | Maternal other AEFI | 2 |
| Lung disorder | Maternal other AEFI | 2 |
| Hyperaesthesia | Maternal other AEFI | 2 |
| Joint range of motion decreased | Maternal other AEFI | 2 |
| Head discomfort | Maternal other AEFI | 2 |
| Blood pressure fluctuation | Maternal other AEFI | 2 |
| Vaccination site lymphadenopathy | Maternal other AEFI | 2 |
| Cardiovascular disorder | Maternal other AEFI | 2 |
| Sneezing | Maternal other AEFI | 2 |
| Mastitis | Maternal other AEFI | 2 |
| Depressed mood | Maternal other AEFI | 2 |
| Vaccination site reaction | Maternal other AEFI | 2 |
| Clavicle fracture | Maternal other AEFI | 2 |
| Pneumonia | Maternal other AEFI | 2 |
| Skin mass | Maternal other AEFI | 2 |
| Balance disorder | Maternal other AEFI | 2 |
| Vaccination site oedema | Maternal other AEFI | 2 |
| Blepharospasm | Maternal other AEFI | 2 |
| Mechanical urticaria | Maternal other AEFI | 2 |
| Human chorionic gonadotropin increased | Maternal other AEFI | 2 |
| Muscle contractions involuntary | Maternal other AEFI | 2 |
| Hallucination | Maternal other AEFI | 2 |
| Breast swelling | Maternal other AEFI | 2 |
| Injection site pruritus | Maternal other AEFI | 2 |
| Petechiae | Maternal other AEFI | 2 |
| Impaired work ability | Maternal other AEFI | 2 |
| Vomiting projectile | Maternal other AEFI | 2 |
| Dyspepsia | Maternal other AEFI | 2 |
| Throat irritation | Maternal other AEFI | 2 |
| Seasonal allergy | Maternal other AEFI | 2 |
| Poor quality sleep | Maternal other AEFI | 2 |
| Mobility decreased | Maternal other AEFI | 2 |
| Injection site warmth | Maternal other AEFI | 2 |
| Musculoskeletal chest pain | Maternal other AEFI | 2 |
| Delivery | Maternal other AEFI | 2 |
| Lower respiratory tract infection | Maternal other AEFI | 2 |
| Stress | Maternal other AEFI | 2 |
| Lip swelling | Maternal other AEFI | 2 |
| Toothache | Maternal other AEFI | 2 |
| Feeling of body temperature change | Maternal other AEFI | 2 |
| Pulmonary pain | Maternal other AEFI | 2 |
| Ovulation delayed | Maternal other AEFI | 2 |
| Blood glucose increased | Maternal other AEFI | 2 |
| Vaccination site discomfort | Maternal other AEFI | 2 |
| Flatulence | Maternal other AEFI | 2 |
| Vaccination complication | Maternal other AEFI | 2 |
| Disorientation | Maternal other AEFI | 2 |
| Fall | Maternal other AEFI | 2 |
| Cardiac disorder | Maternal other AEFI | 2 |
| Swelling face | Maternal other AEFI | 2 |
| Pain in jaw | Maternal other AEFI | 2 |
| Gestational hypertension | Pregnancy, labour and delivery complications and risk factors (excl abortions and stillbirth) | 2 |
| Placental infarction | Pregnancy, labour and delivery complications and risk factors (excl abortions and stillbirth) | 2 |
| Labour complication | Pregnancy, labour and delivery complications and risk factors (excl abortions and stillbirth) | 2 |
| Vulvovaginal injury | Pregnancy, labour and delivery complications and risk factors (excl abortions and stillbirth) | 2 |
| Prolonged labour | Pregnancy, labour and delivery complications and risk factors (excl abortions and stillbirth) | 2 |
| Placental insufficiency | Pregnancy, labour and delivery complications and risk factors (excl abortions and stillbirth) | 2 |
| Hyperemesis gravidarum | Pregnancy, labour and delivery complications and risk factors (excl abortions and stillbirth) | 2 |
| Uterine contractions abnormal | Pregnancy, labour and delivery complications and risk factors (excl abortions and stillbirth) | 2 |
| Complication of delivery | Pregnancy, labour and delivery complications and risk factors (excl abortions and stillbirth) | 2 |
| Benign hydatidiform mole | Pregnancy, labour and delivery complications and risk factors (excl abortions and stillbirth) | 2 |
| Funisitis | Neonatal disorders | 2 |
| Low birth weight baby | Neonatal disorders | 2 |
| Neonatal asphyxia | Neonatal disorders | 2 |
| Meconium aspiration syndrome | Neonatal disorders | 2 |
| Neonatal pneumothorax | Neonatal disorders | 2 |
| Apgar score low | Neonatal disorders | 2 |
| Meconium in amniotic fluid | Foetal disorders | 2 |
| Ultrasound foetal abnormal | Foetal disorders | 2 |
| Hydrops foetalis | Foetal disorders | 2 |
| Foetal cystic hygroma | Foetal disorders | 2 |
| Hydrocephalus | Congenital, familial and genetic disorders | 2 |
| Gastroschisis | Congenital, familial and genetic disorders | 2 |
| Congenital great vessel anomaly | Congenital, familial and genetic disorders | 2 |
| Ventricular hypoplasia | Congenital, familial and genetic disorders | 2 |
| Atrioventricular septal defect | Congenital, familial and genetic disorders | 2 |
| Hypoplastic left heart syndrome | Congenital, familial and genetic disorders | 2 |
| Anencephaly | Congenital, familial and genetic disorders | 2 |
| Cleft lip and palate | Congenital, familial and genetic disorders | 2 |
| Congenital absence of cranial vault | Congenital, familial and genetic disorders | 2 |
| Cytogenetic abnormality | Congenital, familial and genetic disorders | 2 |
| Trisomy 18 | Congenital, familial and genetic disorders | 2 |
| Kidney malformation | Congenital, familial and genetic disorders | 2 |
| Anal fistula | Neonatal other AEFI | 1 |
| Urine sodium abnormal | Neonatal other AEFI | 1 |
| Dyspnoea | Neonatal other AEFI | 1 |
| Sepsis | Neonatal other AEFI | 1 |
| Rash maculo-papular | Neonatal other AEFI | 1 |
| Rash pruritic | Neonatal other AEFI | 1 |
| Hypotonia | Neonatal other AEFI | 1 |
| Neonatal asphyxia | Neonatal other AEFI | 1 |
| Tremor | Neonatal other AEFI | 1 |
| Brain injury | Neonatal other AEFI | 1 |
| Clavicle fracture | Neonatal other AEFI | 1 |
| Dry skin | Neonatal other AEFI | 1 |
| Skin exfoliation | Neonatal other AEFI | 1 |
| Pulmonary arterial pressure abnormal | Neonatal other AEFI | 1 |
| Ventricular hypertrophy | Neonatal other AEFI | 1 |
| Extramedullary haemopoiesis | Neonatal other AEFI | 1 |
| Extremity contracture | Neonatal other AEFI | 1 |
| Joint range of motion decreased | Neonatal other AEFI | 1 |
| Musculoskeletal disorder | Neonatal other AEFI | 1 |
| Scoliosis | Neonatal other AEFI | 1 |
| Visceral oedema | Neonatal other AEFI | 1 |
| Intestinal dilatation | Neonatal other AEFI | 1 |
| Renal failure | Neonatal other AEFI | 1 |
| Generalised oedema | Neonatal other AEFI | 1 |
| Acoustic stimulation tests abnormal | Neonatal other AEFI | 1 |
| Pyelitis | Neonatal other AEFI | 1 |
| Cough | Neonatal other AEFI | 1 |
| Nasopharyngitis | Neonatal other AEFI | 1 |
| Motor developmental delay | Neonatal other AEFI | 1 |
| Muscle tone disorder | Neonatal other AEFI | 1 |
| Rhinorrhoea | Neonatal other AEFI | 1 |
| Sleep disorder | Neonatal other AEFI | 1 |
| Sneezing | Neonatal other AEFI | 1 |
| Inguinal hernia | Neonatal other AEFI | 1 |
| Tachycardia | Neonatal other AEFI | 1 |
| Feeling jittery | Neonatal other AEFI | 1 |
| Pneumothorax | Neonatal other AEFI | 1 |
| Myocarditis | Foetal other AEFI | 1 |
| Pericarditis | Foetal other AEFI | 1 |
| Brain hypoxia | Foetal other AEFI | 1 |
| Lethargy | Foetal other AEFI | 1 |
| Respiratory arrest | Foetal other AEFI | 1 |
| Vasodilatation | Foetal other AEFI | 1 |
| Chromosome analysis abnormal | Foetal other AEFI | 1 |
| Intraventricular haemorrhage | Foetal other AEFI | 1 |
| Venous aneurysm | Foetal other AEFI | 1 |
| Rash maculo-papular | Maternal other AEFI | 1 |
| Rash pustular | Maternal other AEFI | 1 |
| Abdominal infection | Maternal other AEFI | 1 |
| Appendicitis perforated | Maternal other AEFI | 1 |
| Complicated appendicitis | Maternal other AEFI | 1 |
| Dermatosis | Maternal other AEFI | 1 |
| Hypersensitivity vasculitis | Maternal other AEFI | 1 |
| C-reactive protein increased | Maternal other AEFI | 1 |
| Embolism venous | Maternal other AEFI | 1 |
| Osteoarthritis | Maternal other AEFI | 1 |
| Cerebrovascular accident | Maternal other AEFI | 1 |
| Lacrimation increased | Maternal other AEFI | 1 |
| Increased appetite | Maternal other AEFI | 1 |
| Appendicitis | Maternal other AEFI | 1 |
| Hepatic function abnormal | Maternal other AEFI | 1 |
| Renal impairment | Maternal other AEFI | 1 |
| Fibrin D dimer increased | Maternal other AEFI | 1 |
| Sensory disturbance | Maternal other AEFI | 1 |
| Dyskinesia | Maternal other AEFI | 1 |
| Hyperemesis gravidarum | Maternal other AEFI | 1 |
| Thrombosis with thrombocytopenia syndrome | Maternal other AEFI | 1 |
| Unresponsive to stimuli | Maternal other AEFI | 1 |
| General physical health deterioration | Maternal other AEFI | 1 |
| Cholecystitis | Maternal other AEFI | 1 |
| Glomerulonephritis chronic | Maternal other AEFI | 1 |
| Clinically isolated syndrome | Maternal other AEFI | 1 |
| Cyanosis | Maternal other AEFI | 1 |
| Lymphadenitis | Maternal other AEFI | 1 |
| Escherichia bacteraemia | Maternal other AEFI | 1 |
| Somatic symptom disorder | Maternal other AEFI | 1 |
| Tunnel vision | Maternal other AEFI | 1 |
| Emotional disorder | Maternal other AEFI | 1 |
| Temperature regulation disorder | Maternal other AEFI | 1 |
| Thrombocytopenia | Maternal other AEFI | 1 |
| Erythema nodosum | Maternal other AEFI | 1 |
| Varicose veins pelvic | Maternal other AEFI | 1 |
| Nasal herpes | Maternal other AEFI | 1 |
| Anaemia postoperative | Maternal other AEFI | 1 |
| Leukopenia | Maternal other AEFI | 1 |
| Glucose urine present | Maternal other AEFI | 1 |
| Urine ketone body present | Maternal other AEFI | 1 |
| Thrombophlebitis | Maternal other AEFI | 1 |
| Facial paresis | Maternal other AEFI | 1 |
| Hyperventilation | Maternal other AEFI | 1 |
| Obstructive airways disorder | Maternal other AEFI | 1 |
| Epilepsy | Maternal other AEFI | 1 |
| Antinuclear antibody | Maternal other AEFI | 1 |
| CSF protein increased | Maternal other AEFI | 1 |
| CSF white blood cell count increased | Maternal other AEFI | 1 |
| Noninfectious myelitis | Maternal other AEFI | 1 |
| Blood glucose decreased | Maternal other AEFI | 1 |
| Anal sphincter atony | Maternal other AEFI | 1 |
| Generalised tonic-clonic seizure | Maternal other AEFI | 1 |
| Postictal state | Maternal other AEFI | 1 |
| Cognitive disorder | Maternal other AEFI | 1 |
| Local reaction | Maternal other AEFI | 1 |
| Vaccination site hypersensitivity | Maternal other AEFI | 1 |
| Anovulatory cycle | Maternal other AEFI | 1 |
| Disseminated Bacillus Calmette-Guerin infection | Maternal other AEFI | 1 |
| Abdominal rigidity | Maternal other AEFI | 1 |
| Pharyngeal erythema | Maternal other AEFI | 1 |
| Thrombotic thrombocytopenic purpura | Maternal other AEFI | 1 |
| Cholelithiasis | Maternal other AEFI | 1 |
| Cholestasis | Maternal other AEFI | 1 |
| Hyperreflexia | Maternal other AEFI | 1 |
| Xerophthalmia | Maternal other AEFI | 1 |
| Bronchospasm | Maternal other AEFI | 1 |
| Perinatal depression | Maternal other AEFI | 1 |
| Staphylococcal infection | Maternal other AEFI | 1 |
| Protein urine present | Maternal other AEFI | 1 |
| Weight increased | Maternal other AEFI | 1 |
| Corneal reflex decreased | Maternal other AEFI | 1 |
| Mastoid disorder | Maternal other AEFI | 1 |
| Odynophagia | Maternal other AEFI | 1 |
| Vaccination site joint movement impairment | Maternal other AEFI | 1 |
| Ulcer | Maternal other AEFI | 1 |
| Application site pain | Maternal other AEFI | 1 |
| Muscle fatigue | Maternal other AEFI | 1 |
| Eye irritation | Maternal other AEFI | 1 |
| Troponin increased | Maternal other AEFI | 1 |
| Rotator cuff syndrome | Maternal other AEFI | 1 |
| Tachyarrhythmia | Maternal other AEFI | 1 |
| Extrasystoles | Maternal other AEFI | 1 |
| Infarction | Maternal other AEFI | 1 |
| Mood altered | Maternal other AEFI | 1 |
| Agitation | Maternal other AEFI | 1 |
| Heart rate irregular | Maternal other AEFI | 1 |
| Amnesia | Maternal other AEFI | 1 |
| Gingival bleeding | Maternal other AEFI | 1 |
| Puncture site pruritus | Maternal other AEFI | 1 |
| Suicidal behaviour | Maternal other AEFI | 1 |
| Gastrointestinal disorder | Maternal other AEFI | 1 |
| Benign hydatidiform mole | Maternal other AEFI | 1 |
| Migraine with aura | Maternal other AEFI | 1 |
| Injected limb mobility decreased | Maternal other AEFI | 1 |
| Hepatitis | Maternal other AEFI | 1 |
| Pancreatitis | Maternal other AEFI | 1 |
| Suicidal ideation | Maternal other AEFI | 1 |
| Sepsis | Maternal other AEFI | 1 |
| Vaccination site infection | Maternal other AEFI | 1 |
| Bursitis | Maternal other AEFI | 1 |
| Postmenopausal haemorrhage | Maternal other AEFI | 1 |
| Carpal tunnel syndrome | Maternal other AEFI | 1 |
| Ovarian haemorrhage | Maternal other AEFI | 1 |
| Blood urine present | Maternal other AEFI | 1 |
| Skin warm | Maternal other AEFI | 1 |
| Lymphoma | Maternal other AEFI | 1 |
| Joint swelling | Maternal other AEFI | 1 |
| Joint warmth | Maternal other AEFI | 1 |
| Internal haemorrhage | Maternal other AEFI | 1 |
| Progesterone decreased | Maternal other AEFI | 1 |
| Conjunctivitis | Maternal other AEFI | 1 |
| Appetite disorder | Maternal other AEFI | 1 |
| Dermatitis | Maternal other AEFI | 1 |
| Alopecia | Maternal other AEFI | 1 |
| Pharyngitis streptococcal | Maternal other AEFI | 1 |
| Sleep apnoea syndrome | Maternal other AEFI | 1 |
| Tonsillitis | Maternal other AEFI | 1 |
| Sudden hearing loss | Maternal other AEFI | 1 |
| Eye oedema | Maternal other AEFI | 1 |
| Macular oedema | Maternal other AEFI | 1 |
| Injection site induration | Maternal other AEFI | 1 |
| Musculoskeletal discomfort | Maternal other AEFI | 1 |
| Skin burning sensation | Maternal other AEFI | 1 |
| Premenstrual syndrome | Maternal other AEFI | 1 |
| Autonomic nervous system imbalance | Maternal other AEFI | 1 |
| Postural orthostatic tachycardia syndrome | Maternal other AEFI | 1 |
| Sensation of foreign body | Maternal other AEFI | 1 |
| Gastric disorder | Maternal other AEFI | 1 |
| Haematosalpinx | Maternal other AEFI | 1 |
| Arrhythmia | Maternal other AEFI | 1 |
| Rectal haemorrhage | Maternal other AEFI | 1 |
| Kidney infection | Maternal other AEFI | 1 |
| International normalised ratio abnormal | Maternal other AEFI | 1 |
| Oral herpes | Maternal other AEFI | 1 |
| Vaccination site induration | Maternal other AEFI | 1 |
| Vulvovaginal pain | Maternal other AEFI | 1 |
| Proteinuria | Maternal other AEFI | 1 |
| Sweating fever | Maternal other AEFI | 1 |
| Vaccination site urticaria | Maternal other AEFI | 1 |
| Ventricular extrasystoles | Maternal other AEFI | 1 |
| Bradycardia | Maternal other AEFI | 1 |
| Ketonuria | Maternal other AEFI | 1 |
| Lymph node abscess | Maternal other AEFI | 1 |
| Candida infection | Maternal other AEFI | 1 |
| Areflexia | Maternal other AEFI | 1 |
| Road traffic accident | Maternal other AEFI | 1 |
| Coagulopathy | Maternal other AEFI | 1 |
| Procedural nausea | Maternal other AEFI | 1 |
| Uterine tenderness | Maternal other AEFI | 1 |
| Blood loss anaemia | Maternal other AEFI | 1 |
| Allergy to vaccine | Maternal other AEFI | 1 |
| Breast inflammation | Maternal other AEFI | 1 |
| Suppressed lactation | Maternal other AEFI | 1 |
| Fallopian tube disorder | Maternal other AEFI | 1 |
| Haemoperitoneum | Maternal other AEFI | 1 |
| Endometriosis | Maternal other AEFI | 1 |
| Gastroenteritis | Maternal other AEFI | 1 |
| Dermatitis atopic | Maternal other AEFI | 1 |
| Tongue blistering | Maternal other AEFI | 1 |
| Polymenorrhoea | Maternal other AEFI | 1 |
| Productive cough | Maternal other AEFI | 1 |
| Pain of skin | Maternal other AEFI | 1 |
| Oral discomfort | Maternal other AEFI | 1 |
| Eating disorder | Maternal other AEFI | 1 |
| Multiple sclerosis relapse | Maternal other AEFI | 1 |
| Depersonalisation/derealisation disorder | Maternal other AEFI | 1 |
| Vaginal ulceration | Maternal other AEFI | 1 |
| Nerve injury | Maternal other AEFI | 1 |
| Ear infection | Maternal other AEFI | 1 |
| Aphthous ulcer | Maternal other AEFI | 1 |
| Renal colic | Maternal other AEFI | 1 |
| Iridocyclitis | Maternal other AEFI | 1 |
| Diplopia | Maternal other AEFI | 1 |
| Persistent postural-perceptual dizziness | Maternal other AEFI | 1 |
| Retinal vascular disorder | Maternal other AEFI | 1 |
| Visual field defect | Maternal other AEFI | 1 |
| Periarthritis | Maternal other AEFI | 1 |
| Tendon pain | Maternal other AEFI | 1 |
| Anger | Maternal other AEFI | 1 |
| Mental disorder | Maternal other AEFI | 1 |
| Injection site reaction | Maternal other AEFI | 1 |
| Tendonitis | Maternal other AEFI | 1 |
| Sinus headache | Maternal other AEFI | 1 |
| Haematemesis | Maternal other AEFI | 1 |
| Papule | Maternal other AEFI | 1 |
| Post-traumatic neck syndrome | Maternal other AEFI | 1 |
| Ovulation pain | Maternal other AEFI | 1 |
| Nasal obstruction | Maternal other AEFI | 1 |
| Sinus congestion | Maternal other AEFI | 1 |
| Glossodynia | Maternal other AEFI | 1 |
| Tongue ulceration | Maternal other AEFI | 1 |
| Haemorrhoids | Maternal other AEFI | 1 |
| Feeding disorder | Maternal other AEFI | 1 |
| Injection site discomfort | Maternal other AEFI | 1 |
| Abscess limb | Maternal other AEFI | 1 |
| Panic attack | Maternal other AEFI | 1 |
| Choking | Maternal other AEFI | 1 |
| Infertility | Maternal other AEFI | 1 |
| Contusion | Maternal other AEFI | 1 |
| Platelet count decreased | Maternal other AEFI | 1 |
| Terminal insomnia | Maternal other AEFI | 1 |
| Status epilepticus | Maternal other AEFI | 1 |
| Immune thrombocytopenia | Maternal other AEFI | 1 |
| Traumatic haematoma | Maternal other AEFI | 1 |
| Heart rate | Maternal other AEFI | 1 |
| Vein disorder | Maternal other AEFI | 1 |
| Impaired gastric emptying | Maternal other AEFI | 1 |
| Administration site pain | Maternal other AEFI | 1 |
| Myositis | Maternal other AEFI | 1 |
| Bradykinesia | Maternal other AEFI | 1 |
| Renal failure | Maternal other AEFI | 1 |
| Gingival pain | Maternal other AEFI | 1 |
| Mouth swelling | Maternal other AEFI | 1 |
| Oral mucosal erythema | Maternal other AEFI | 1 |
| Hyperglycaemia | Maternal other AEFI | 1 |
| Hyperthyroidism | Maternal other AEFI | 1 |
| Meniere's disease | Maternal other AEFI | 1 |
| Vaccination site pruritus | Maternal other AEFI | 1 |
| Symphysiolysis | Maternal other AEFI | 1 |
| Urinary incontinence | Maternal other AEFI | 1 |
| Chillblains | Maternal other AEFI | 1 |
| Infectious mononucleosis | Maternal other AEFI | 1 |
| Flank pain | Maternal other AEFI | 1 |
| Dizziness exertional | Maternal other AEFI | 1 |
| Body temperature abnormal | Maternal other AEFI | 1 |
| Groin pain | Maternal other AEFI | 1 |
| Hypopnoea | Maternal other AEFI | 1 |
| Psychomotor hyperactivity | Maternal other AEFI | 1 |
| Restlessness | Maternal other AEFI | 1 |
| Breast tenderness | Maternal other AEFI | 1 |
| Painful respiration | Maternal other AEFI | 1 |
| Pulpitis dental | Maternal other AEFI | 1 |
| Skin reaction | Maternal other AEFI | 1 |
| Bone pain | Maternal other AEFI | 1 |
| Dry throat | Maternal other AEFI | 1 |
| Injury | Maternal other AEFI | 1 |
| Lip haemorrhage | Maternal other AEFI | 1 |
| Ear discomfort | Maternal other AEFI | 1 |
| Histamine level increased | Maternal other AEFI | 1 |
| Cervix disorder | Maternal other AEFI | 1 |
| Pityriasis rosea | Maternal other AEFI | 1 |
| Body temperature fluctuation | Maternal other AEFI | 1 |
| Inferior vena caval occlusion | Maternal other AEFI | 1 |
| Muscle tightness | Maternal other AEFI | 1 |
| Application site haematoma | Maternal other AEFI | 1 |
| Cystitis | Maternal other AEFI | 1 |
| Cytomegalovirus infection reactivation | Maternal other AEFI | 1 |
| Coma scale abnormal | Maternal other AEFI | 1 |
| Tonic convulsion | Maternal other AEFI | 1 |
| Ovarian cyst | Maternal other AEFI | 1 |
| Toxocariasis | Maternal other AEFI | 1 |
| Toxoplasma serology positive | Maternal other AEFI | 1 |
| Hypoglycaemia | Maternal other AEFI | 1 |
| Asymptomatic COVID-19 | Maternal other AEFI | 1 |
| Blindness | Maternal other AEFI | 1 |
| Adnexa uteri pain | Maternal other AEFI | 1 |
| Galactostasis | Maternal other AEFI | 1 |
| Thyroid cancer | Maternal other AEFI | 1 |
| Angioedema | Maternal other AEFI | 1 |
| Ascites | Maternal other AEFI | 1 |
| Parosmia | Maternal other AEFI | 1 |
| Orthopnoea | Maternal other AEFI | 1 |
| Taste disorder | Maternal other AEFI | 1 |
| Oedema peripheral | Maternal other AEFI | 1 |
| Heart rate decreased | Maternal other AEFI | 1 |
| Sjogren's syndrome | Maternal other AEFI | 1 |
| Hormone level abnormal | Maternal other AEFI | 1 |
| Colour blindness | Maternal other AEFI | 1 |
| Hemiplegia | Maternal other AEFI | 1 |
| Head banging | Maternal other AEFI | 1 |
| Depression | Maternal other AEFI | 1 |
| Vitamin D decreased | Maternal other AEFI | 1 |
| Deafness unilateral | Maternal other AEFI | 1 |
| Oral pruritus | Maternal other AEFI | 1 |
| Breast mass | Maternal other AEFI | 1 |
| Premature baby death | Termination of pregnancy and risk of abortion | 1 |
| Abortion threatened | Termination of pregnancy and risk of abortion | 1 |
| Abortion spontaneous complicated | Termination of pregnancy and risk of abortion | 1 |
| Abortion incomplete | Termination of pregnancy and risk of abortion | 1 |
| Death | Termination of pregnancy and risk of abortion | 1 |
| Mastitis | Pregnancy, labour and delivery complications and risk factors (excl abortions and stillbirth) | 1 |
| Placental calcification | Pregnancy, labour and delivery complications and risk factors (excl abortions and stillbirth) | 1 |
| Retroplacental haematoma | Pregnancy, labour and delivery complications and risk factors (excl abortions and stillbirth) | 1 |
| Puerperal pyrexia | Pregnancy, labour and delivery complications and risk factors (excl abortions and stillbirth) | 1 |
| Prolonged pregnancy | Pregnancy, labour and delivery complications and risk factors (excl abortions and stillbirth) | 1 |
| Small size placenta | Pregnancy, labour and delivery complications and risk factors (excl abortions and stillbirth) | 1 |
| Glucose tolerance impaired in pregnancy | Pregnancy, labour and delivery complications and risk factors (excl abortions and stillbirth) | 1 |
| Amniorrhoea | Pregnancy, labour and delivery complications and risk factors (excl abortions and stillbirth) | 1 |
| Uterine irritability | Pregnancy, labour and delivery complications and risk factors (excl abortions and stillbirth) | 1 |
| Induced labour | Pregnancy, labour and delivery complications and risk factors (excl abortions and stillbirth) | 1 |
| Herpes gestationis | Pregnancy, labour and delivery complications and risk factors (excl abortions and stillbirth) | 1 |
| Intrapartum haemorrhage | Pregnancy, labour and delivery complications and risk factors (excl abortions and stillbirth) | 1 |
| Gestational trophoblastic detachment | Pregnancy, labour and delivery complications and risk factors (excl abortions and stillbirth) | 1 |
| Intrauterine infection | Pregnancy, labour and delivery complications and risk factors (excl abortions and stillbirth) | 1 |
| Uterine hypertonus | Pregnancy, labour and delivery complications and risk factors (excl abortions and stillbirth) | 1 |
| Subchorionic haemorrhage | Pregnancy, labour and delivery complications and risk factors (excl abortions and stillbirth) | 1 |
| Uterine hypotonus | Pregnancy, labour and delivery complications and risk factors (excl abortions and stillbirth) | 1 |
| Shoulder dystocia | Pregnancy, labour and delivery complications and risk factors (excl abortions and stillbirth) | 1 |
| Premature baby | Pregnancy, labour and delivery complications and risk factors (excl abortions and stillbirth) | 1 |
| Retained placenta or membranes | Pregnancy, labour and delivery complications and risk factors (excl abortions and stillbirth) | 1 |
| Placenta praevia | Pregnancy, labour and delivery complications and risk factors (excl abortions and stillbirth) | 1 |
| Necrotising enterocolitis neonatal | Neonatal disorders | 1 |
| Neonatal seizure | Neonatal disorders | 1 |
| Perinatal stroke | Neonatal disorders | 1 |
| Ventricular septal defect | Neonatal disorders | 1 |
| Hyperbilirubinaemia neonatal | Neonatal disorders | 1 |
| Weight decrease neonatal | Neonatal disorders | 1 |
| Hypothermia neonatal | Neonatal disorders | 1 |
| Infantile apnoea | Neonatal disorders | 1 |
| Neutropenia neonatal | Neonatal disorders | 1 |
| Respiratory disorder neonatal | Neonatal disorders | 1 |
| Neonatal respiratory failure | Neonatal disorders | 1 |
| Sepsis neonatal | Neonatal disorders | 1 |
| Neonatal tachypnoea | Neonatal disorders | 1 |
| Cardiomyopathy neonatal | Neonatal disorders | 1 |
| Small for dates baby | Neonatal disorders | 1 |
| Polyhydramnios | Foetal disorders | 1 |
| Amniotic cavity infection | Foetal disorders | 1 |
| Maternal condition affecting foetus | Foetal disorders | 1 |
| Foetal disorder | Foetal disorders | 1 |
| Cerebral haemorrhage foetal | Foetal disorders | 1 |
| Foetal heart rate acceleration abnormality | Foetal disorders | 1 |
| Foetal damage | Foetal disorders | 1 |
| Anencephaly | Foetal disorders | 1 |
| Kidney duplex | Foetal disorders | 1 |
| Cerebral infarction foetal | Foetal disorders | 1 |
| Foetal heart rate increased | Foetal disorders | 1 |
| Umbilical cord around neck | Foetal disorders | 1 |
| Foetal chromosome abnormality | Foetal disorders | 1 |
| Trisomy 13 | Congenital, familial and genetic disorders | 1 |
| Encephalocele | Congenital, familial and genetic disorders | 1 |
| Hydronephrosis | Congenital, familial and genetic disorders | 1 |
| Congenital hydrocephalus | Congenital, familial and genetic disorders | 1 |
| Spina bifida | Congenital, familial and genetic disorders | 1 |
| Nail aplasia | Congenital, familial and genetic disorders | 1 |
| Congenital renal disorder | Congenital, familial and genetic disorders | 1 |
| Hydranencephaly | Congenital, familial and genetic disorders | 1 |
| Fallot's tetralogy | Congenital, familial and genetic disorders | 1 |
| Double outlet right ventricle | Congenital, familial and genetic disorders | 1 |
| Congenital aortic stenosis | Congenital, familial and genetic disorders | 1 |
| Congenital eye disorder | Congenital, familial and genetic disorders | 1 |
| Dysmorphism | Congenital, familial and genetic disorders | 1 |
| Single umbilical artery | Congenital, familial and genetic disorders | 1 |
| Urethral valves | Congenital, familial and genetic disorders | 1 |
| Sturge-Weber syndrome | Congenital, familial and genetic disorders | 1 |
| Aberrant aortic arch | Congenital, familial and genetic disorders | 1 |
| Macrocephaly | Congenital, familial and genetic disorders | 1 |
| PTEN gene mutation | Congenital, familial and genetic disorders | 1 |
| Cleft palate | Congenital, familial and genetic disorders | 1 |
| Ductus arteriosus premature closure | Congenital, familial and genetic disorders | 1 |
| Trisomy 8 | Congenital, familial and genetic disorders | 1 |
| Aorta hypoplasia | Congenital, familial and genetic disorders | 1 |
| Univentricular heart | Congenital, familial and genetic disorders | 1 |
| Anophthalmos | Congenital, familial and genetic disorders | 1 |
| Tremor | Congenital, familial and genetic disorders | 1 |
| Renal dysplasia | Congenital, familial and genetic disorders | 1 |
| Pulmonary malformation | Congenital, familial and genetic disorders | 1 |
| Syndactyly | Congenital, familial and genetic disorders | 1 |
| Wolf-Hirschhorn syndrome | Congenital, familial and genetic disorders | 1 |
| Tourette's disorder | Congenital, familial and genetic disorders | 1 |
| Porencephaly | Congenital, familial and genetic disorders | 1 |

**B) Moderna vaccine**

| **PTs** | **SMQ** | **N** |
| --- | --- | --- |
| Abortion spontaneous | Termination of pregnancy and risk of abortion | 326 |
| Fatigue | Maternal other AEFI | 136 |
| Pyrexia | Maternal other AEFI | 133 |
| Headache | Maternal other AEFI | 122 |
| Pain in extremity | Maternal other AEFI | 93 |
| Chills | Maternal other AEFI | 74 |
| Myalgia | Maternal other AEFI | 71 |
| Nausea | Maternal other AEFI | 70 |
| Pain | Maternal other AEFI | 56 |
| Vaginal haemorrhage | Maternal other AEFI | 54 |
| Foetal death | Termination of pregnancy and risk of abortion | 45 |
| Dizziness | Maternal other AEFI | 44 |
| Vomiting | Maternal other AEFI | 41 |
| Malaise | Maternal other AEFI | 40 |
| Arthralgia | Maternal other AEFI | 37 |
| Abdominal pain | Maternal other AEFI | 33 |
| Vaccination site pain | Maternal other AEFI | 31 |
| Haemorrhage in pregnancy | Pregnancy, labour and delivery complications and risk factors (excl abortions and stillbirth) | 31 |
| Injection site pain | Maternal other AEFI | 28 |
| Dyspnoea | Maternal other AEFI | 26 |
| Premature labour | Pregnancy, labour and delivery complications and risk factors (excl abortions and stillbirth) | 26 |
| Haemorrhage | Maternal other AEFI | 23 |
| Premature delivery | Pregnancy, labour and delivery complications and risk factors (excl abortions and stillbirth) | 23 |
| Syncope | Maternal other AEFI | 22 |
| Pruritus | Maternal other AEFI | 19 |
| Induced labour | Pregnancy, labour and delivery complications and risk factors (excl abortions and stillbirth) | 19 |
| Delivery | Maternal other AEFI | 17 |
| Peripheral swelling | Maternal other AEFI | 16 |
| Lymphadenopathy | Maternal other AEFI | 16 |
| Muscle spasms | Maternal other AEFI | 16 |
| Pre-eclampsia | Pregnancy, labour and delivery complications and risk factors (excl abortions and stillbirth) | 16 |
| Foetal hypokinesia | Foetal disorders | 16 |
| Back pain | Maternal other AEFI | 15 |
| Injection site erythema | Maternal other AEFI | 15 |
| Rash | Maternal other AEFI | 15 |
| Stillbirth | Termination of pregnancy and risk of abortion | 15 |
| Chest pain | Maternal other AEFI | 14 |
| Diarrhoea | Maternal other AEFI | 14 |
| Illness | Maternal other AEFI | 14 |
| Erythema | Maternal other AEFI | 14 |
| Abortion missed | Termination of pregnancy and risk of abortion | 14 |
| Foetal heart rate abnormal | Foetal disorders | 14 |
| Thrombosis | Maternal other AEFI | 13 |
| Uterine contractions during pregnancy | Pregnancy, labour and delivery complications and risk factors (excl abortions and stillbirth) | 13 |
| Influenza like illness | Maternal other AEFI | 12 |
| Paraesthesia | Maternal other AEFI | 12 |
| Premature rupture of membranes | Pregnancy, labour and delivery complications and risk factors (excl abortions and stillbirth) | 12 |
| Premature baby | Neonatal disorders | 12 |
| Foetal growth restriction | Foetal disorders | 12 |
| Asthenia | Maternal other AEFI | 11 |
| Injection site swelling | Maternal other AEFI | 11 |
| Caesarean section | Pregnancy, labour and delivery complications and risk factors (excl abortions and stillbirth) | 11 |
| Abdominal pain upper | Maternal other AEFI | 10 |
| Decreased appetite | Maternal other AEFI | 10 |
| Palpitations | Maternal other AEFI | 10 |
| Rash erythematous | Maternal other AEFI | 10 |
| Deep vein thrombosis | Maternal other AEFI | 10 |
| Injection site inflammation | Maternal other AEFI | 10 |
| Premature separation of placenta | Pregnancy, labour and delivery complications and risk factors (excl abortions and stillbirth) | 10 |
| Migraine | Maternal other AEFI | 9 |
| Anxiety | Maternal other AEFI | 9 |
| Feeling abnormal | Maternal other AEFI | 9 |
| Injection site pruritus | Maternal other AEFI | 9 |
| Vaginal discharge | Maternal other AEFI | 9 |
| Insomnia | Maternal other AEFI | 9 |
| Preterm premature rupture of membranes | Pregnancy, labour and delivery complications and risk factors (excl abortions and stillbirth) | 9 |
| Heart rate increased | Maternal other AEFI | 8 |
| Vaccination site erythema | Maternal other AEFI | 8 |
| Heavy menstrual bleeding | Maternal other AEFI | 8 |
| Intermenstrual bleeding | Maternal other AEFI | 8 |
| Nasopharyngitis | Maternal other AEFI | 8 |
| COVID-19 | Maternal other AEFI | 8 |
| Influenza | Maternal other AEFI | 8 |
| Vaccination site rash | Maternal other AEFI | 8 |
| Loss of consciousness | Maternal other AEFI | 8 |
| Lethargy | Maternal other AEFI | 8 |
| Abortion induced | Termination of pregnancy and risk of abortion | 8 |
| Anembryonic gestation | Termination of pregnancy and risk of abortion | 8 |
| Gestational diabetes | Pregnancy, labour and delivery complications and risk factors (excl abortions and stillbirth) | 8 |
| Ectopic pregnancy | Pregnancy, labour and delivery complications and risk factors (excl abortions and stillbirth) | 8 |
| Tachycardia | Maternal other AEFI | 7 |
| Neck pain | Maternal other AEFI | 7 |
| Musculoskeletal stiffness | Maternal other AEFI | 7 |
| Cough | Maternal other AEFI | 7 |
| Chest discomfort | Maternal other AEFI | 7 |
| Hyperhidrosis | Maternal other AEFI | 7 |
| Injection site warmth | Maternal other AEFI | 7 |
| Vaccination site pruritus | Maternal other AEFI | 7 |
| Postpartum haemorrhage | Pregnancy, labour and delivery complications and risk factors (excl abortions and stillbirth) | 7 |
| Hypertension | Maternal other AEFI | 6 |
| Pain of skin | Maternal other AEFI | 6 |
| Vaccination site swelling | Maternal other AEFI | 6 |
| Hypotension | Maternal other AEFI | 6 |
| Anaemia | Maternal other AEFI | 6 |
| Hypoaesthesia | Maternal other AEFI | 6 |
| Rash pruritic | Maternal other AEFI | 6 |
| Night sweats | Maternal other AEFI | 6 |
| Vaccination complication | Maternal other AEFI | 6 |
| Asymptomatic COVID-19 | Maternal other AEFI | 6 |
| Foetal disorder | Foetal disorders | 6 |
| Amniorrhoea | Foetal disorders | 6 |
| Uterine dilation and curettage | Maternal other AEFI | 5 |
| Tremor | Maternal other AEFI | 5 |
| Injection site reaction | Maternal other AEFI | 5 |
| Skin reaction | Maternal other AEFI | 5 |
| Contusion | Maternal other AEFI | 5 |
| Tinnitus | Maternal other AEFI | 5 |
| Feeling hot | Maternal other AEFI | 5 |
| Abortion | Termination of pregnancy and risk of abortion | 5 |
| Polyhydramnios | Foetal disorders | 5 |
| Tenderness | Maternal other AEFI | 4 |
| Menstruation irregular | Maternal other AEFI | 4 |
| Blood pressure increased | Maternal other AEFI | 4 |
| Injection site rash | Maternal other AEFI | 4 |
| Dysmenorrhoea | Maternal other AEFI | 4 |
| Muscular weakness | Maternal other AEFI | 4 |
| Seizure | Maternal other AEFI | 4 |
| Cervical dilatation | Maternal other AEFI | 4 |
| Skin warm | Maternal other AEFI | 4 |
| Axillary pain | Maternal other AEFI | 4 |
| Swelling face | Maternal other AEFI | 4 |
| Vaccination site warmth | Maternal other AEFI | 4 |
| Lymph node pain | Maternal other AEFI | 4 |
| Thrombocytopenia | Maternal other AEFI | 4 |
| Discomfort | Maternal other AEFI | 4 |
| Abdominal discomfort | Maternal other AEFI | 4 |
| Pulmonary embolism | Maternal other AEFI | 4 |
| Tension headache | Maternal other AEFI | 4 |
| Swelling | Maternal other AEFI | 4 |
| Blood pressure decreased | Maternal other AEFI | 4 |
| Gestational hypertension | Pregnancy, labour and delivery complications and risk factors (excl abortions and stillbirth) | 4 |
| Ultrasound antenatal screen abnormal | Foetal disorders | 4 |
| Rash macular | Maternal other AEFI | 3 |
| Visual impairment | Maternal other AEFI | 3 |
| Neuralgia | Maternal other AEFI | 3 |
| Pelvic pain | Maternal other AEFI | 3 |
| Feeling cold | Maternal other AEFI | 3 |
| Breech presentation | Maternal other AEFI | 3 |
| Menstrual disorder | Maternal other AEFI | 3 |
| Menstruation delayed | Maternal other AEFI | 3 |
| Somnolence | Maternal other AEFI | 3 |
| Injected limb mobility decreased | Maternal other AEFI | 3 |
| Nasal congestion | Maternal other AEFI | 3 |
| Rash papular | Maternal other AEFI | 3 |
| Sleep disorder | Maternal other AEFI | 3 |
| Erythema multiforme | Maternal other AEFI | 3 |
| Urticaria | Maternal other AEFI | 3 |
| Myocarditis | Maternal other AEFI | 3 |
| Cerebrovascular accident | Maternal other AEFI | 3 |
| Anticoagulant therapy | Maternal other AEFI | 3 |
| Swollen tongue | Maternal other AEFI | 3 |
| Drug ineffective | Maternal other AEFI | 3 |
| Condition aggravated | Maternal other AEFI | 3 |
| Hot flush | Maternal other AEFI | 3 |
| Vertigo | Maternal other AEFI | 3 |
| Impaired work ability | Maternal other AEFI | 3 |
| Bone pain | Maternal other AEFI | 3 |
| Fall | Maternal other AEFI | 3 |
| Uterine haemorrhage | Maternal other AEFI | 3 |
| Muscle tightness | Maternal other AEFI | 3 |
| Mobility decreased | Maternal other AEFI | 3 |
| Cellulitis | Maternal other AEFI | 3 |
| Abortion threatened | Termination of pregnancy and risk of abortion | 3 |
| Retained placenta or membranes | Pregnancy, labour and delivery complications and risk factors (excl abortions and stillbirth) | 3 |
| Placenta praevia | Pregnancy, labour and delivery complications and risk factors (excl abortions and stillbirth) | 3 |
| Placental disorder | Pregnancy, labour and delivery complications and risk factors (excl abortions and stillbirth) | 3 |
| Subchorionic haematoma | Pregnancy, labour and delivery complications and risk factors (excl abortions and stillbirth) | 3 |
| Amniotic fluid index decreased | Pregnancy, labour and delivery complications and risk factors (excl abortions and stillbirth) | 3 |
| Subchorionic haemorrhage | Pregnancy, labour and delivery complications and risk factors (excl abortions and stillbirth) | 3 |
| Low birth weight baby | Neonatal disorders | 3 |
| Neonatal aspiration | Neonatal disorders | 3 |
| Foetal movement disorder | Foetal disorders | 3 |
| Hydrops foetalis | Foetal disorders | 3 |
| Supraventricular extrasystoles | Maternal other AEFI | 2 |
| Ventricular extrasystoles | Maternal other AEFI | 2 |
| Presyncope | Maternal other AEFI | 2 |
| Burning sensation | Maternal other AEFI | 2 |
| Pericarditis | Maternal other AEFI | 2 |
| Pleuritic pain | Maternal other AEFI | 2 |
| Vision blurred | Maternal other AEFI | 2 |
| Infection | Maternal other AEFI | 2 |
| General physical health deterioration | Maternal other AEFI | 2 |
| Hyperaesthesia | Maternal other AEFI | 2 |
| Metabolic function test | Maternal other AEFI | 2 |
| Heart rate | Maternal other AEFI | 2 |
| Multigravida | Maternal other AEFI | 2 |
| Facial paralysis | Maternal other AEFI | 2 |
| Dyskinesia | Maternal other AEFI | 2 |
| Ovarian cyst | Maternal other AEFI | 2 |
| Pulmonary oedema | Maternal other AEFI | 2 |
| Bell's palsy | Maternal other AEFI | 2 |
| Mental fatigue | Maternal other AEFI | 2 |
| Odynophagia | Maternal other AEFI | 2 |
| Unresponsive to stimuli | Maternal other AEFI | 2 |
| Bedridden | Maternal other AEFI | 2 |
| Uterine spasm | Maternal other AEFI | 2 |
| Dehydration | Maternal other AEFI | 2 |
| Injection site urticaria | Maternal other AEFI | 2 |
| Uterine contractions during pregnancy | Maternal other AEFI | 2 |
| Pneumonia | Maternal other AEFI | 2 |
| Aphasia | Maternal other AEFI | 2 |
| Neurological symptom | Maternal other AEFI | 2 |
| Gait inability | Maternal other AEFI | 2 |
| Pharyngeal swelling | Maternal other AEFI | 2 |
| Morning sickness | Maternal other AEFI | 2 |
| Injection site discomfort | Maternal other AEFI | 2 |
| Amenorrhoea | Maternal other AEFI | 2 |
| Vaccination site induration | Maternal other AEFI | 2 |
| Hypersensitivity | Maternal other AEFI | 2 |
| Ageusia | Maternal other AEFI | 2 |
| Depression | Maternal other AEFI | 2 |
| Head discomfort | Maternal other AEFI | 2 |
| Dry mouth | Maternal other AEFI | 2 |
| Migraine with aura | Maternal other AEFI | 2 |
| Asthma | Maternal other AEFI | 2 |
| Gait disturbance | Maternal other AEFI | 2 |
| Immune thrombocytopenia | Maternal other AEFI | 2 |
| Acute hepatic failure | Maternal other AEFI | 2 |
| Epilepsy | Maternal other AEFI | 2 |
| Feeling of body temperature change | Maternal other AEFI | 2 |
| Oropharyngeal pain | Maternal other AEFI | 2 |
| Injection site mass | Maternal other AEFI | 2 |
| Ear pain | Maternal other AEFI | 2 |
| Herpes zoster | Maternal other AEFI | 2 |
| Hypokinesia | Maternal other AEFI | 2 |
| Dyspepsia | Maternal other AEFI | 2 |
| Disseminated intravascular coagulation | Maternal other AEFI | 2 |
| Anaphylactic reaction | Maternal other AEFI | 2 |
| Tongue blistering | Maternal other AEFI | 2 |
| Lip swelling | Maternal other AEFI | 2 |
| Prolonged labour | Pregnancy, labour and delivery complications and risk factors (excl abortions and stillbirth) | 2 |
| Uterine atony | Pregnancy, labour and delivery complications and risk factors (excl abortions and stillbirth) | 2 |
| Uterine contractions abnormal | Pregnancy, labour and delivery complications and risk factors (excl abortions and stillbirth) | 2 |
| High risk pregnancy | Pregnancy, labour and delivery complications and risk factors (excl abortions and stillbirth) | 2 |
| Death neonatal | Neonatal disorders | 2 |
| Ultrasound foetal abnormal | Foetal disorders | 2 |
| Umbilical cord abnormality | Foetal disorders | 2 |
| Foetal heart rate disorder | Foetal disorders | 2 |
| Foetal malformation | Foetal disorders | 2 |
| Foetal heart rate decreased | Foetal disorders | 2 |
| Foetal heart rate increased | Foetal disorders | 2 |
| Umbilical cord prolapse | Foetal disorders | 2 |
| Tachycardia foetal | Foetal disorders | 2 |
| Atrial septal defect | Congenital, familial and genetic disorders | 2 |
| Multiple congenital abnormalities | Congenital, familial and genetic disorders | 2 |
| Ventricular septal defect | Congenital, familial and genetic disorders | 2 |
| Conjoined twins | Congenital, familial and genetic disorders | 2 |
| Patent ductus arteriosus | Congenital, familial and genetic disorders | 2 |
| Cleft palate | Congenital, familial and genetic disorders | 2 |
| Cardiac dysfunction | Neonatal other AEFI | 1 |
| Oxygen therapy | Neonatal other AEFI | 1 |
| Fatigue | Neonatal other AEFI | 1 |
| Obstructive airways disorder | Neonatal other AEFI | 1 |
| Weight gain poor | Neonatal other AEFI | 1 |
| Respiratory distress | Neonatal other AEFI | 1 |
| Coombs positive haemolytic anaemia | Neonatal other AEFI | 1 |
| Haemolysis | Neonatal other AEFI | 1 |
| Arrhythmia | Maternal other AEFI | 1 |
| Allergy to vaccine | Maternal other AEFI | 1 |
| Proctalgia | Maternal other AEFI | 1 |
| Vaccination site haematoma | Maternal other AEFI | 1 |
| Polymenorrhoea | Maternal other AEFI | 1 |
| Amphetamines negative | Maternal other AEFI | 1 |
| Anion gap | Maternal other AEFI | 1 |
| Barbiturates negative | Maternal other AEFI | 1 |
| Cytomegalovirus test positive | Maternal other AEFI | 1 |
| Magnetic resonance imaging abdominal abnormal | Maternal other AEFI | 1 |
| Atelectasis | Maternal other AEFI | 1 |
| Pulmonary calcification | Maternal other AEFI | 1 |
| Blood glucose increased | Maternal other AEFI | 1 |
| Blood test abnormal | Maternal other AEFI | 1 |
| Hepatic enzyme increased | Maternal other AEFI | 1 |
| Computerised tomogram abdomen | Maternal other AEFI | 1 |
| Computerised tomogram abdomen abnormal | Maternal other AEFI | 1 |
| Laboratory test abnormal | Maternal other AEFI | 1 |
| Ultrasound scan abnormal | Maternal other AEFI | 1 |
| Herpes ophthalmic | Maternal other AEFI | 1 |
| Blister | Maternal other AEFI | 1 |
| Dyshidrotic eczema | Maternal other AEFI | 1 |
| Hypopnoea | Maternal other AEFI | 1 |
| Respiration abnormal | Maternal other AEFI | 1 |
| Oligomenorrhoea | Maternal other AEFI | 1 |
| Blindness | Maternal other AEFI | 1 |
| Breast pain | Maternal other AEFI | 1 |
| Cognitive disorder | Maternal other AEFI | 1 |
| Confusional state | Maternal other AEFI | 1 |
| Formication | Maternal other AEFI | 1 |
| Lyme disease | Maternal other AEFI | 1 |
| Tongue ulceration | Maternal other AEFI | 1 |
| Hyperbilirubinaemia | Maternal other AEFI | 1 |
| Nephrolithiasis | Maternal other AEFI | 1 |
| Sepsis | Maternal other AEFI | 1 |
| Blood bilirubin increased | Maternal other AEFI | 1 |
| Blood glucose decreased | Maternal other AEFI | 1 |
| Uterine dilation and evacuation | Maternal other AEFI | 1 |
| Photophobia | Maternal other AEFI | 1 |
| COVID-19 pneumonia | Maternal other AEFI | 1 |
| Vaccination failure | Maternal other AEFI | 1 |
| Increased appetite | Maternal other AEFI | 1 |
| Taste disorder | Maternal other AEFI | 1 |
| Blood thyroid stimulating hormone | Maternal other AEFI | 1 |
| Cardiac monitoring | Maternal other AEFI | 1 |
| Echocardiogram | Maternal other AEFI | 1 |
| Supraventricular tachycardia | Maternal other AEFI | 1 |
| Ventricular tachycardia | Maternal other AEFI | 1 |
| Rhinorrhoea | Maternal other AEFI | 1 |
| Endometritis | Maternal other AEFI | 1 |
| Cerebral haemorrhage | Maternal other AEFI | 1 |
| Cervical discharge | Maternal other AEFI | 1 |
| Cervix disorder | Maternal other AEFI | 1 |
| Paralysis | Maternal other AEFI | 1 |
| Vaccine breakthrough infection | Maternal other AEFI | 1 |
| Petechiae | Maternal other AEFI | 1 |
| Eye irritation | Maternal other AEFI | 1 |
| Oral discomfort | Maternal other AEFI | 1 |
| Visual field defect | Maternal other AEFI | 1 |
| Body temperature increased | Maternal other AEFI | 1 |
| Blood pressure fluctuation | Maternal other AEFI | 1 |
| C-reactive protein increased | Maternal other AEFI | 1 |
| Oxygen saturation decreased | Maternal other AEFI | 1 |
| Respiratory rate increased | Maternal other AEFI | 1 |
| Systemic lupus erythematosus | Maternal other AEFI | 1 |
| Multisystem inflammatory syndrome in adults | Maternal other AEFI | 1 |
| Oropharyngeal discomfort | Maternal other AEFI | 1 |
| Hemianaesthesia | Maternal other AEFI | 1 |
| Leukocytosis | Maternal other AEFI | 1 |
| Perinatal depression | Maternal other AEFI | 1 |
| Vaccination site mass | Maternal other AEFI | 1 |
| Vaccination site discomfort | Maternal other AEFI | 1 |
| Face oedema | Maternal other AEFI | 1 |
| Oedema mucosal | Maternal other AEFI | 1 |
| Coagulopathy | Maternal other AEFI | 1 |
| Emotional distress | Maternal other AEFI | 1 |
| Jaw disorder | Maternal other AEFI | 1 |
| Scar | Maternal other AEFI | 1 |
| Balance disorder | Maternal other AEFI | 1 |
| Guillain-Barre syndrome | Maternal other AEFI | 1 |
| Musculoskeletal pain | Maternal other AEFI | 1 |
| Limb discomfort | Maternal other AEFI | 1 |
| Breast feeding | Maternal other AEFI | 1 |
| Caesarean section | Maternal other AEFI | 1 |
| Laparotomy | Maternal other AEFI | 1 |
| Postpartum state | Maternal other AEFI | 1 |
| Coronary artery dissection | Maternal other AEFI | 1 |
| Myocardial infarction | Maternal other AEFI | 1 |
| Normal labour | Maternal other AEFI | 1 |
| Renal pain | Maternal other AEFI | 1 |
| Coma | Maternal other AEFI | 1 |
| Red blood cell count decreased | Maternal other AEFI | 1 |
| White blood cell count decreased | Maternal other AEFI | 1 |
| Adenomyosis | Maternal other AEFI | 1 |
| Fallopian tube perforation | Maternal other AEFI | 1 |
| Lymphoedema | Maternal other AEFI | 1 |
| Pregnancy | Maternal other AEFI | 1 |
| Neuropathy peripheral | Maternal other AEFI | 1 |
| Ovarian disorder | Maternal other AEFI | 1 |
| Psychotic disorder | Maternal other AEFI | 1 |
| Miliaria | Maternal other AEFI | 1 |
| Sensitive skin | Maternal other AEFI | 1 |
| Diabetes mellitus inadequate control | Maternal other AEFI | 1 |
| Prophylaxis of nausea and vomiting | Maternal other AEFI | 1 |
| Cardiac dysfunction | Maternal other AEFI | 1 |
| Cardiac failure congestive | Maternal other AEFI | 1 |
| Intensive care | Maternal other AEFI | 1 |
| Mechanical ventilation | Maternal other AEFI | 1 |
| Haematoma | Maternal other AEFI | 1 |
| Bundle branch block | Maternal other AEFI | 1 |
| Oropharyngeal blistering | Maternal other AEFI | 1 |
| Macrosomia | Maternal other AEFI | 1 |
| Bacterial infection | Maternal other AEFI | 1 |
| Sweating fever | Maternal other AEFI | 1 |
| Incorrect dose administered | Maternal other AEFI | 1 |
| Pain in jaw | Maternal other AEFI | 1 |
| Sinus pain | Maternal other AEFI | 1 |
| Trismus | Maternal other AEFI | 1 |
| Laryngitis | Maternal other AEFI | 1 |
| Ear discomfort | Maternal other AEFI | 1 |
| Hypoacusis | Maternal other AEFI | 1 |
| Cold sweat | Maternal other AEFI | 1 |
| Pancreatitis acute | Maternal other AEFI | 1 |
| Cardiolipin antibody positive | Maternal other AEFI | 1 |
| Eye pain | Maternal other AEFI | 1 |
| Sleep deficit | Maternal other AEFI | 1 |
| Haemoptysis | Maternal other AEFI | 1 |
| Lymphadenitis | Maternal other AEFI | 1 |
| Myofascial pain syndrome | Maternal other AEFI | 1 |
| Atrial fibrillation | Maternal other AEFI | 1 |
| Fear | Maternal other AEFI | 1 |
| Acute hepatitis B | Maternal other AEFI | 1 |
| Chromaturia | Maternal other AEFI | 1 |
| Hypoglycaemia | Maternal other AEFI | 1 |
| Jaundice | Maternal other AEFI | 1 |
| Lactic acidosis | Maternal other AEFI | 1 |
| Ocular icterus | Maternal other AEFI | 1 |
| Septic shock | Maternal other AEFI | 1 |
| Tachypnoea | Maternal other AEFI | 1 |
| Mastitis | Maternal other AEFI | 1 |
| Pollakiuria | Maternal other AEFI | 1 |
| Urinary tract pain | Maternal other AEFI | 1 |
| Food aversion | Maternal other AEFI | 1 |
| Terminal insomnia | Maternal other AEFI | 1 |
| Dry throat | Maternal other AEFI | 1 |
| Injection site haematoma | Maternal other AEFI | 1 |
| Pyelocaliectasis | Maternal other AEFI | 1 |
| Muscle twitching | Maternal other AEFI | 1 |
| Eyelid ptosis | Maternal other AEFI | 1 |
| Lacrimation increased | Maternal other AEFI | 1 |
| Pregnancy on oral contraceptive | Maternal other AEFI | 1 |
| Weight decreased | Maternal other AEFI | 1 |
| Feeling jittery | Maternal other AEFI | 1 |
| Memory impairment | Maternal other AEFI | 1 |
| Near death experience | Maternal other AEFI | 1 |
| Regurgitation | Maternal other AEFI | 1 |
| Sneezing | Maternal other AEFI | 1 |
| Nodule | Maternal other AEFI | 1 |
| Haemorrhoids | Maternal other AEFI | 1 |
| Vulvovaginal candidiasis | Maternal other AEFI | 1 |
| Protein urine present | Maternal other AEFI | 1 |
| Angina pectoris | Maternal other AEFI | 1 |
| Inflammation | Maternal other AEFI | 1 |
| Screaming | Maternal other AEFI | 1 |
| Procedural haemorrhage | Maternal other AEFI | 1 |
| Uterine leiomyoma | Maternal other AEFI | 1 |
| Impaired healing | Maternal other AEFI | 1 |
| Vaccination site hypoaesthesia | Maternal other AEFI | 1 |
| Facial spasm | Maternal other AEFI | 1 |
| Gynaecological examination abnormal | Maternal other AEFI | 1 |
| Smear vaginal normal | Maternal other AEFI | 1 |
| Ultrasound abdomen abnormal | Maternal other AEFI | 1 |
| Haematosalpinx | Maternal other AEFI | 1 |
| First trimester pregnancy | Maternal other AEFI | 1 |
| Induration | Maternal other AEFI | 1 |
| Skin hyperpigmentation | Maternal other AEFI | 1 |
| Malnutrition | Maternal other AEFI | 1 |
| Pancreatitis | Maternal other AEFI | 1 |
| Discharge | Maternal other AEFI | 1 |
| Skin swelling | Maternal other AEFI | 1 |
| Adverse reaction | Maternal other AEFI | 1 |
| Breech delivery | Maternal other AEFI | 1 |
| Acute myocardial infarction | Maternal other AEFI | 1 |
| Anaesthesia oral | Maternal other AEFI | 1 |
| Hemiparesis | Maternal other AEFI | 1 |
| Hypotonia | Maternal other AEFI | 1 |
| Pulseless electrical activity | Maternal other AEFI | 1 |
| Crohn's disease | Maternal other AEFI | 1 |
| Acute kidney injury | Maternal other AEFI | 1 |
| Bradycardia | Maternal other AEFI | 1 |
| Hyponatraemia | Maternal other AEFI | 1 |
| Haematuria | Maternal other AEFI | 1 |
| Haemorrhage urinary tract | Maternal other AEFI | 1 |
| Micturition urgency | Maternal other AEFI | 1 |
| Loss of personal independence in daily activities | Maternal other AEFI | 1 |
| Vaccination site movement impairment | Maternal other AEFI | 1 |
| Dysphagia | Maternal other AEFI | 1 |
| Cardio-respiratory arrest | Maternal other AEFI | 1 |
| Cholecystitis acute | Maternal other AEFI | 1 |
| Cholecystitis infective | Maternal other AEFI | 1 |
| Cholelithiasis | Maternal other AEFI | 1 |
| Hydrocholecystis | Maternal other AEFI | 1 |
| Fluid retention | Maternal other AEFI | 1 |
| Glossodynia | Maternal other AEFI | 1 |
| Lip injury | Maternal other AEFI | 1 |
| Tongue discomfort | Maternal other AEFI | 1 |
| Tongue erythema | Maternal other AEFI | 1 |
| Weight increased | Maternal other AEFI | 1 |
| Blood loss anaemia | Maternal other AEFI | 1 |
| Haemorrhagic ovarian cyst | Maternal other AEFI | 1 |
| Ovarian cyst ruptured | Maternal other AEFI | 1 |
| Skin atrophy | Maternal other AEFI | 1 |
| Artificial insemination | Maternal other AEFI | 1 |
| Candida infection | Maternal other AEFI | 1 |
| Drug reaction with eosinophilia and systemic symptoms | Maternal other AEFI | 1 |
| Eosinophilia | Maternal other AEFI | 1 |
| Lung infiltration | Maternal other AEFI | 1 |
| Non-cardiac chest pain | Maternal other AEFI | 1 |
| Aortic dissection | Maternal other AEFI | 1 |
| Chlamydial infection | Maternal other AEFI | 1 |
| Gonorrhoea | Maternal other AEFI | 1 |
| Depressed mood | Maternal other AEFI | 1 |
| Developmental delay | Maternal other AEFI | 1 |
| Stress | Maternal other AEFI | 1 |
| Pemphigoid | Maternal other AEFI | 1 |
| Blood disorder | Maternal other AEFI | 1 |
| Productive cough | Maternal other AEFI | 1 |
| Injection site infection | Maternal other AEFI | 1 |
| Autoimmune hepatitis | Maternal other AEFI | 1 |
| Hepatitis C | Maternal other AEFI | 1 |
| Hepatomegaly | Maternal other AEFI | 1 |
| Ophthalmic migraine | Maternal other AEFI | 1 |
| Kidney infection | Maternal other AEFI | 1 |
| Groin pain | Maternal other AEFI | 1 |
| Fluorescent in situ hybridisation | Maternal other AEFI | 1 |
| Haematoma infection | Maternal other AEFI | 1 |
| Sinus tachycardia | Maternal other AEFI | 1 |
| Conjunctival haemorrhage | Maternal other AEFI | 1 |
| Cardiac flutter | Maternal other AEFI | 1 |
| Vaccination site reaction | Maternal other AEFI | 1 |
| Body temperature fluctuation | Maternal other AEFI | 1 |
| Oral mucosal blistering | Maternal other AEFI | 1 |
| Foetal hypokinesia | Maternal other AEFI | 1 |
| Clavicle fracture | Maternal other AEFI | 1 |
| Hemiparaesthesia | Maternal other AEFI | 1 |
| Dysgeusia | Maternal other AEFI | 1 |
| Flushing | Maternal other AEFI | 1 |
| Reduced facial expression | Maternal other AEFI | 1 |
| Shock | Maternal other AEFI | 1 |
| Erythema nodosum | Maternal other AEFI | 1 |
| Ovarian cancer | Maternal other AEFI | 1 |
| Injection site induration | Maternal other AEFI | 1 |
| Induced abortion failed | Termination of pregnancy and risk of abortion | 1 |
| Abortion spontaneous incomplete | Termination of pregnancy and risk of abortion | 1 |
| Biochemical pregnancy | Termination of pregnancy and risk of abortion | 1 |
| Abortion of ectopic pregnancy | Termination of pregnancy and risk of abortion | 1 |
| Ectopic pregnancy termination | Pregnancy, labour and delivery complications and risk factors (excl abortions and stillbirth) | 1 |
| Precipitate labour | Pregnancy, labour and delivery complications and risk factors (excl abortions and stillbirth) | 1 |
| HELLP syndrome | Pregnancy, labour and delivery complications and risk factors (excl abortions and stillbirth) | 1 |
| Cholestasis of pregnancy | Pregnancy, labour and delivery complications and risk factors (excl abortions and stillbirth) | 1 |
| Labour complication | Pregnancy, labour and delivery complications and risk factors (excl abortions and stillbirth) | 1 |
| Postpartum disorder | Pregnancy, labour and delivery complications and risk factors (excl abortions and stillbirth) | 1 |
| Threatened labour | Pregnancy, labour and delivery complications and risk factors (excl abortions and stillbirth) | 1 |
| Labour induction | Pregnancy, labour and delivery complications and risk factors (excl abortions and stillbirth) | 1 |
| Acute fatty liver of pregnancy | Pregnancy, labour and delivery complications and risk factors (excl abortions and stillbirth) | 1 |
| Perineal injury | Pregnancy, labour and delivery complications and risk factors (excl abortions and stillbirth) | 1 |
| Uterine hypertonus | Pregnancy, labour and delivery complications and risk factors (excl abortions and stillbirth) | 1 |
| Cervical incompetence | Pregnancy, labour and delivery complications and risk factors (excl abortions and stillbirth) | 1 |
| Placental insufficiency | Pregnancy, labour and delivery complications and risk factors (excl abortions and stillbirth) | 1 |
| Peripartum cardiomyopathy | Pregnancy, labour and delivery complications and risk factors (excl abortions and stillbirth) | 1 |
| Abnormal cord insertion | Pregnancy, labour and delivery complications and risk factors (excl abortions and stillbirth) | 1 |
| Eclampsia | Pregnancy, labour and delivery complications and risk factors (excl abortions and stillbirth) | 1 |
| Amniotic membrane rupture test positive | Pregnancy, labour and delivery complications and risk factors (excl abortions and stillbirth) | 1 |
| Ruptured ectopic pregnancy | Pregnancy, labour and delivery complications and risk factors (excl abortions and stillbirth) | 1 |
| Prolonged pregnancy | Pregnancy, labour and delivery complications and risk factors (excl abortions and stillbirth) | 1 |
| Placental calcification | Pregnancy, labour and delivery complications and risk factors (excl abortions and stillbirth) | 1 |
| Failed induction of labour | Pregnancy, labour and delivery complications and risk factors (excl abortions and stillbirth) | 1 |
| Shortened cervix | Pregnancy, labour and delivery complications and risk factors (excl abortions and stillbirth) | 1 |
| Complication of pregnancy | Pregnancy, labour and delivery complications and risk factors (excl abortions and stillbirth) | 1 |
| Placenta praevia haemorrhage | Pregnancy, labour and delivery complications and risk factors (excl abortions and stillbirth) | 1 |
| Shoulder dystocia | Pregnancy, labour and delivery complications and risk factors (excl abortions and stillbirth) | 1 |
| Hypoglycaemia neonatal | Neonatal disorders | 1 |
| Hyperbilirubinaemia neonatal | Neonatal disorders | 1 |
| Neutropenia neonatal | Neonatal disorders | 1 |
| Meconium aspiration syndrome | Neonatal disorders | 1 |
| Neonatal respiratory distress | Neonatal disorders | 1 |
| Jaundice neonatal | Neonatal disorders | 1 |
| Neonatal dyspnoea | Neonatal disorders | 1 |
| Cerebral haemorrhage neonatal | Neonatal disorders | 1 |
| Hypertension neonatal | Neonatal disorders | 1 |
| Neonatal disorder | Neonatal disorders | 1 |
| Pulmonary oedema neonatal | Neonatal disorders | 1 |
| Respiratory disorder neonatal | Neonatal disorders | 1 |
| Foetal growth abnormality | Foetal disorders | 1 |
| Foetal arrhythmia | Foetal disorders | 1 |
| Oligohydramnios | Foetal disorders | 1 |
| Foetal cerebrovascular disorder | Foetal disorders | 1 |
| Umbilical cord around neck | Foetal disorders | 1 |
| Amniotic cavity disorder | Foetal disorders | 1 |
| Amniotic fluid volume increased | Foetal disorders | 1 |
| Amniotic cavity infection | Foetal disorders | 1 |
| Biopsy chorionic villous abnormal | Foetal disorders | 1 |
| Foetal renal impairment | Foetal disorders | 1 |
| Foetal chromosome abnormality | Foetal disorders | 1 |
| Foetal cardiac disorder | Foetal disorders | 1 |
| Foetal macrosomia | Foetal disorders | 1 |
| Bradycardia foetal | Foetal disorders | 1 |
| Anencephaly | Congenital, familial and genetic disorders | 1 |
| Eyelid ptosis congenital | Congenital, familial and genetic disorders | 1 |
| Kidney duplex | Congenital, familial and genetic disorders | 1 |
| Marcus Gunn syndrome | Congenital, familial and genetic disorders | 1 |
| Exomphalos | Congenital, familial and genetic disorders | 1 |
| Spinal disorder | Congenital, familial and genetic disorders | 1 |
| Coloboma | Congenital, familial and genetic disorders | 1 |
| Laryngomalacia | Congenital, familial and genetic disorders | 1 |
| Oesophageal atresia | Congenital, familial and genetic disorders | 1 |
| Tracheo-oesophageal fistula | Congenital, familial and genetic disorders | 1 |
| Fallot's tetralogy | Congenital, familial and genetic disorders | 1 |
| Trisomy 22 | Congenital, familial and genetic disorders | 1 |
| Renal aplasia | Congenital, familial and genetic disorders | 1 |
| Congenital hydronephrosis | Congenital, familial and genetic disorders | 1 |
| Congenital skin disorder | Congenital, familial and genetic disorders | 1 |
| Cleft lip | Congenital, familial and genetic disorders | 1 |
| Congenital diaphragmatic hernia | Congenital, familial and genetic disorders | 1 |
| Hypoplastic left heart syndrome | Congenital, familial and genetic disorders | 1 |
| Cleft uvula | Congenital, familial and genetic disorders | 1 |
| Congenital nose malformation | Congenital, familial and genetic disorders | 1 |
| Cytogenetic abnormality | Congenital, familial and genetic disorders | 1 |
| Limb malformation | Congenital, familial and genetic disorders | 1 |
| Pyelocaliectasis | Congenital, familial and genetic disorders | 1 |
| Haemoglobinopathy | Congenital, familial and genetic disorders | 1 |

**C) Oxford-AstraZeneca vaccine**

| **PTs** | **SMQ** | **N** |
| --- | --- | --- |
| Abortion spontaneous | Termination of pregnancy and risk of abortion | 222 |
| Pyrexia | Maternal other AEFI | 145 |
| Headache | Maternal other AEFI | 139 |
| Myalgia | Maternal other AEFI | 91 |
| Chills | Maternal other AEFI | 80 |
| Fatigue | Maternal other AEFI | 79 |
| Arthralgia | Maternal other AEFI | 48 |
| Nausea | Maternal other AEFI | 46 |
| Pain | Maternal other AEFI | 43 |
| Asthenia | Maternal other AEFI | 35 |
| Pain in extremity | Maternal other AEFI | 31 |
| Vomiting | Maternal other AEFI | 30 |
| Dizziness | Maternal other AEFI | 30 |
| Vaginal haemorrhage | Maternal other AEFI | 23 |
| Malaise | Maternal other AEFI | 17 |
| Tenderness | Maternal other AEFI | 17 |
| Abortion | Termination of pregnancy and risk of abortion | 17 |
| Abdominal pain | Maternal other AEFI | 16 |
| Dyspnoea | Maternal other AEFI | 16 |
| Hyperhidrosis | Maternal other AEFI | 15 |
| Injection site pain | Maternal other AEFI | 14 |
| Muscle spasms | Maternal other AEFI | 14 |
| Chest pain | Maternal other AEFI | 13 |
| Decreased appetite | Maternal other AEFI | 13 |
| Cough | Maternal other AEFI | 13 |
| Heavy menstrual bleeding | Maternal other AEFI | 13 |
| Haemorrhage | Maternal other AEFI | 13 |
| Migraine | Maternal other AEFI | 13 |
| Tremor | Maternal other AEFI | 13 |
| Illness | Maternal other AEFI | 12 |
| COVID-19 | Maternal other AEFI | 11 |
| Palpitations | Maternal other AEFI | 11 |
| Influenza like illness | Maternal other AEFI | 10 |
| Body temperature increased | Maternal other AEFI | 10 |
| Hypoaesthesia | Maternal other AEFI | 10 |
| Tachycardia | Maternal other AEFI | 10 |
| Diarrhoea | Maternal other AEFI | 10 |
| Menstruation delayed | Maternal other AEFI | 9 |
| Oropharyngeal pain | Maternal other AEFI | 9 |
| Thrombosis | Maternal other AEFI | 9 |
| Abdominal pain upper | Maternal other AEFI | 8 |
| Peripheral swelling | Maternal other AEFI | 8 |
| Back pain | Maternal other AEFI | 8 |
| Rash | Maternal other AEFI | 8 |
| Paraesthesia | Maternal other AEFI | 8 |
| Night sweats | Maternal other AEFI | 7 |
| Neck pain | Maternal other AEFI | 7 |
| Gestational diabetes | Pregnancy, labour and delivery complications and risk factors (excl abortions and stillbirth) | 7 |
| Deep vein thrombosis | Maternal other AEFI | 6 |
| Pruritus | Maternal other AEFI | 6 |
| Contusion | Maternal other AEFI | 6 |
| Application site pain | Maternal other AEFI | 6 |
| Seizure | Maternal other AEFI | 6 |
| Vaccination site pain | Maternal other AEFI | 6 |
| Pain in jaw | Maternal other AEFI | 6 |
| Limb discomfort | Maternal other AEFI | 6 |
| Erythema | Maternal other AEFI | 6 |
| Somnolence | Maternal other AEFI | 6 |
| Insomnia | Maternal other AEFI | 6 |
| Premature baby | Neonatal disorders | 5 |
| Rhinorrhoea | Maternal other AEFI | 5 |
| Swelling | Maternal other AEFI | 5 |
| Vision blurred | Maternal other AEFI | 5 |
| Lymphadenopathy | Maternal other AEFI | 5 |
| Pulmonary embolism | Maternal other AEFI | 5 |
| Pre-eclampsia | Pregnancy, labour and delivery complications and risk factors (excl abortions and stillbirth) | 5 |
| Premature labour | Pregnancy, labour and delivery complications and risk factors (excl abortions and stillbirth) | 5 |
| Abortion missed | Termination of pregnancy and risk of abortion | 5 |
| Tachycardia foetal | Foetal disorders | 4 |
| Feeling of body temperature change | Maternal other AEFI | 4 |
| Muscular weakness | Maternal other AEFI | 4 |
| Thrombocytopenia | Maternal other AEFI | 4 |
| Photophobia | Maternal other AEFI | 4 |
| Nasal congestion | Maternal other AEFI | 4 |
| Dysmenorrhoea | Maternal other AEFI | 4 |
| Menstruation irregular | Maternal other AEFI | 4 |
| Irritability | Maternal other AEFI | 4 |
| Ectopic pregnancy | Pregnancy, labour and delivery complications and risk factors (excl abortions and stillbirth) | 4 |
| Stillbirth | Termination of pregnancy and risk of abortion | 4 |
| Spina bifida | Congenital, familial and genetic disorders | 3 |
| Foetal vascular malperfusion | Foetal disorders | 3 |
| Menstrual disorder | Maternal other AEFI | 3 |
| Heart rate increased | Maternal other AEFI | 3 |
| Hemiparesis | Maternal other AEFI | 3 |
| Hypertension | Maternal other AEFI | 3 |
| Loss of consciousness | Maternal other AEFI | 3 |
| Ear pain | Maternal other AEFI | 3 |
| Guillain-Barre syndrome | Maternal other AEFI | 3 |
| Paralysis | Maternal other AEFI | 3 |
| Respiratory distress | Maternal other AEFI | 3 |
| Crying | Maternal other AEFI | 3 |
| Urinary tract infection | Maternal other AEFI | 3 |
| Uterine haemorrhage | Maternal other AEFI | 3 |
| Inflammation | Maternal other AEFI | 3 |
| Oedema | Maternal other AEFI | 3 |
| SARS-CoV-2 test positive | Maternal other AEFI | 3 |
| Sensory disturbance | Maternal other AEFI | 3 |
| Musculoskeletal stiffness | Maternal other AEFI | 3 |
| Syncope | Maternal other AEFI | 3 |
| Asthma | Maternal other AEFI | 3 |
| Thirst | Maternal other AEFI | 3 |
| Influenza | Maternal other AEFI | 3 |
| Hypokinesia | Maternal other AEFI | 3 |
| Bone pain | Maternal other AEFI | 3 |
| Hypersensitivity | Maternal other AEFI | 3 |
| Restlessness | Maternal other AEFI | 3 |
| Premature delivery | Pregnancy, labour and delivery complications and risk factors (excl abortions and stillbirth) | 3 |
| Uterine contractions abnormal | Pregnancy, labour and delivery complications and risk factors (excl abortions and stillbirth) | 3 |
| Threatened labour | Pregnancy, labour and delivery complications and risk factors (excl abortions and stillbirth) | 3 |
| Premature rupture of membranes | Pregnancy, labour and delivery complications and risk factors (excl abortions and stillbirth) | 3 |
| Caesarean section | Pregnancy, labour and delivery complications and risk factors (excl abortions and stillbirth) | 3 |
| Abortion induced | Termination of pregnancy and risk of abortion | 3 |
| Trisomy 18 | Congenital, familial and genetic disorders | 2 |
| Coarctation of the aorta | Congenital, familial and genetic disorders | 2 |
| Foetal malformation | Foetal disorders | 2 |
| Perinatal stroke | Neonatal disorders | 2 |
| Low birth weight baby | Neonatal disorders | 2 |
| Cerebrovascular accident | Neonatal other AEFI | 2 |
| Cerebral venous sinus thrombosis | Maternal other AEFI | 2 |
| Thrombosis with thrombocytopenia syndrome | Maternal other AEFI | 2 |
| Pelvic pain | Maternal other AEFI | 2 |
| Hypomenorrhoea | Maternal other AEFI | 2 |
| Blood pressure decreased | Maternal other AEFI | 2 |
| Heart rate abnormal | Maternal other AEFI | 2 |
| Bedridden | Maternal other AEFI | 2 |
| Head discomfort | Maternal other AEFI | 2 |
| Nerve injury | Maternal other AEFI | 2 |
| Gait disturbance | Maternal other AEFI | 2 |
| Muscle strength abnormal | Maternal other AEFI | 2 |
| Petechiae | Maternal other AEFI | 2 |
| Induration | Maternal other AEFI | 2 |
| Nodule | Maternal other AEFI | 2 |
| Anaemia | Maternal other AEFI | 2 |
| Hyperaesthesia | Maternal other AEFI | 2 |
| Uterine spasm | Maternal other AEFI | 2 |
| Discomfort | Maternal other AEFI | 2 |
| Vaginal discharge | Maternal other AEFI | 2 |
| Polymenorrhoea | Maternal other AEFI | 2 |
| Sinus tachycardia | Maternal other AEFI | 2 |
| Haemorrhage urinary tract | Maternal other AEFI | 2 |
| Chest discomfort | Maternal other AEFI | 2 |
| Lethargy | Maternal other AEFI | 2 |
| Anosmia | Maternal other AEFI | 2 |
| Nasopharyngitis | Maternal other AEFI | 2 |
| Amenorrhoea | Maternal other AEFI | 2 |
| Condition aggravated | Maternal other AEFI | 2 |
| Feeling cold | Maternal other AEFI | 2 |
| Dehydration | Maternal other AEFI | 2 |
| Extrasystoles | Maternal other AEFI | 2 |
| Abdominal distension | Maternal other AEFI | 2 |
| Blood pressure increased | Maternal other AEFI | 2 |
| Urticaria | Maternal other AEFI | 2 |
| Hot flush | Maternal other AEFI | 2 |
| Cold sweat | Maternal other AEFI | 2 |
| Rash macular | Maternal other AEFI | 2 |
| Body temperature | Maternal other AEFI | 2 |
| Confusional state | Maternal other AEFI | 2 |
| Paraesthesia oral | Maternal other AEFI | 2 |
| Anaphylactic reaction | Maternal other AEFI | 2 |
| Myocarditis | Maternal other AEFI | 2 |
| Atrial flutter | Maternal other AEFI | 2 |
| Premature separation of placenta | Pregnancy, labour and delivery complications and risk factors (excl abortions and stillbirth) | 2 |
| Haemorrhage in pregnancy | Pregnancy, labour and delivery complications and risk factors (excl abortions and stillbirth) | 2 |
| Hyperemesis gravidarum | Pregnancy, labour and delivery complications and risk factors (excl abortions and stillbirth) | 2 |
| Preterm premature rupture of membranes | Pregnancy, labour and delivery complications and risk factors (excl abortions and stillbirth) | 2 |
| Foetal death | Termination of pregnancy and risk of abortion | 2 |
| Death | Termination of pregnancy and risk of abortion | 2 |
| Anencephaly | Congenital, familial and genetic disorders | 1 |
| Neural tube defect | Congenital, familial and genetic disorders | 1 |
| Ventricular septal defect | Congenital, familial and genetic disorders | 1 |
| Congenital anomaly | Congenital, familial and genetic disorders | 1 |
| Multiple congenital abnormalities | Congenital, familial and genetic disorders | 1 |
| Hydrocephalus | Congenital, familial and genetic disorders | 1 |
| Congenital midline defect | Congenital, familial and genetic disorders | 1 |
| Haemorrhage foetal | Foetal disorders | 1 |
| Foetal distress syndrome | Foetal disorders | 1 |
| Amniorrhoea | Foetal disorders | 1 |
| Foetal heart rate abnormal | Foetal disorders | 1 |
| Umbilical cord around neck | Foetal disorders | 1 |
| Neonatal dyspnoea | Neonatal disorders | 1 |
| Death neonatal | Neonatal disorders | 1 |
| Growth retardation | Neonatal other AEFI | 1 |
| Brain herniation | Maternal other AEFI | 1 |
| Cerebral haemorrhage | Maternal other AEFI | 1 |
| Coma | Maternal other AEFI | 1 |
| Intracranial pressure increased | Maternal other AEFI | 1 |
| Vasogenic cerebral oedema | Maternal other AEFI | 1 |
| Abdominal pain lower | Maternal other AEFI | 1 |
| Throat irritation | Maternal other AEFI | 1 |
| Normal newborn | Maternal other AEFI | 1 |
| Poor quality sleep | Maternal other AEFI | 1 |
| Intermenstrual bleeding | Maternal other AEFI | 1 |
| Hypoacusis | Maternal other AEFI | 1 |
| Increased appetite | Maternal other AEFI | 1 |
| Morning sickness | Maternal other AEFI | 1 |
| Protein urine present | Maternal other AEFI | 1 |
| Weight decreased | Maternal other AEFI | 1 |
| Akinesia | Maternal other AEFI | 1 |
| Facial paresis | Maternal other AEFI | 1 |
| Blood glucose abnormal | Maternal other AEFI | 1 |
| Vertigo | Maternal other AEFI | 1 |
| Amnesia | Maternal other AEFI | 1 |
| Mass | Maternal other AEFI | 1 |
| Brain death | Maternal other AEFI | 1 |
| Haemorrhage intracranial | Maternal other AEFI | 1 |
| Presyncope | Maternal other AEFI | 1 |
| Haemorrhagic stroke | Maternal other AEFI | 1 |
| Vaccination site rash | Maternal other AEFI | 1 |
| Axonal and demyelinating polyneuropathy | Maternal other AEFI | 1 |
| Dysarthria | Maternal other AEFI | 1 |
| Dysphagia | Maternal other AEFI | 1 |
| Epilepsy | Maternal other AEFI | 1 |
| Febrile convulsion | Maternal other AEFI | 1 |
| Generalised tonic-clonic seizure | Maternal other AEFI | 1 |
| Nocturnal dyspnoea | Maternal other AEFI | 1 |
| Thrombotic thrombocytopenic purpura | Maternal other AEFI | 1 |
| Breast pain | Maternal other AEFI | 1 |
| Respiratory failure | Maternal other AEFI | 1 |
| Diplegia | Maternal other AEFI | 1 |
| Meningitis | Maternal other AEFI | 1 |
| Flushing | Maternal other AEFI | 1 |
| Pneumonia | Maternal other AEFI | 1 |
| Secretion discharge | Maternal other AEFI | 1 |
| Sensation of foreign body | Maternal other AEFI | 1 |
| Ovulation delayed | Maternal other AEFI | 1 |
| Vulvovaginal pain | Maternal other AEFI | 1 |
| Restless legs syndrome | Maternal other AEFI | 1 |
| Sinus headache | Maternal other AEFI | 1 |
| Hepatoblastoma | Maternal other AEFI | 1 |
| Hypertrophic cardiomyopathy | Maternal other AEFI | 1 |
| Migraine with aura | Maternal other AEFI | 1 |
| Sweating fever | Maternal other AEFI | 1 |
| Eye pain | Maternal other AEFI | 1 |
| Angina pectoris | Maternal other AEFI | 1 |
| Ageusia | Maternal other AEFI | 1 |
| Abdominal adhesions | Maternal other AEFI | 1 |
| Acute abdomen | Maternal other AEFI | 1 |
| Biliary colic | Maternal other AEFI | 1 |
| Cholecystitis | Maternal other AEFI | 1 |
| Cholelithiasis | Maternal other AEFI | 1 |
| Endometriosis | Maternal other AEFI | 1 |
| Screaming | Maternal other AEFI | 1 |
| Epistaxis | Maternal other AEFI | 1 |
| Gender dysphoria | Maternal other AEFI | 1 |
| Balance disorder | Maternal other AEFI | 1 |
| Anaphylactic shock | Maternal other AEFI | 1 |
| Dry mouth | Maternal other AEFI | 1 |
| Throat tightness | Maternal other AEFI | 1 |
| Scleritis | Maternal other AEFI | 1 |
| Cellulitis | Maternal other AEFI | 1 |
| Acute kidney injury | Maternal other AEFI | 1 |
| Cerebrovascular accident | Maternal other AEFI | 1 |
| Facial paralysis | Maternal other AEFI | 1 |
| Basedow's disease | Maternal other AEFI | 1 |
| Hyperthyroidism | Maternal other AEFI | 1 |
| Premature ovulation | Maternal other AEFI | 1 |
| Cerebral haematoma | Maternal other AEFI | 1 |
| Neurological decompensation | Maternal other AEFI | 1 |
| Oedema peripheral | Maternal other AEFI | 1 |
| Purpura | Maternal other AEFI | 1 |
| Streptococcal infection | Maternal other AEFI | 1 |
| Vascular pain | Maternal other AEFI | 1 |
| Deafness neurosensory | Maternal other AEFI | 1 |
| Sudden hearing loss | Maternal other AEFI | 1 |
| SUNCT syndrome | Maternal other AEFI | 1 |
| Coordination abnormal | Maternal other AEFI | 1 |
| Vaccination site swelling | Maternal other AEFI | 1 |
| Pericarditis | Maternal other AEFI | 1 |
| Skin warm | Maternal other AEFI | 1 |
| Haematochezia | Maternal other AEFI | 1 |
| Hypovolaemic shock | Maternal other AEFI | 1 |
| Muscle strain | Maternal other AEFI | 1 |
| Cervicitis | Maternal other AEFI | 1 |
| Photopsia | Maternal other AEFI | 1 |
| Stevens-Johnson syndrome | Maternal other AEFI | 1 |
| Gait inability | Maternal other AEFI | 1 |
| Hepatic pain | Maternal other AEFI | 1 |
| Sciatica | Maternal other AEFI | 1 |
| Injection site swelling | Maternal other AEFI | 1 |
| Hemiplegic migraine | Maternal other AEFI | 1 |
| Transient ischaemic attack | Maternal other AEFI | 1 |
| Nervousness | Maternal other AEFI | 1 |
| Other ADR | Maternal other AEFI | 1 |
| Feeling abnormal | Maternal other AEFI | 1 |
| Cluster headache | Maternal other AEFI | 1 |
| Developmental delay | Maternal other AEFI | 1 |
| Renal pain | Maternal other AEFI | 1 |
| Typical aura without headache | Maternal other AEFI | 1 |
| Varicophlebitis | Maternal other AEFI | 1 |
| Acne | Maternal other AEFI | 1 |
| Pulmonary pain | Maternal other AEFI | 1 |
| Ovarian cyst | Maternal other AEFI | 1 |
| Bronchitis | Maternal other AEFI | 1 |
| Discharge | Maternal other AEFI | 1 |
| Immunosuppression | Maternal other AEFI | 1 |
| Infection | Maternal other AEFI | 1 |
| Retching | Maternal other AEFI | 1 |
| Sinusitis | Maternal other AEFI | 1 |
| Coagulopathy | Maternal other AEFI | 1 |
| Haematoma | Maternal other AEFI | 1 |
| Pain of skin | Maternal other AEFI | 1 |
| Non-cardiac chest pain | Maternal other AEFI | 1 |
| Abdominal tenderness | Maternal other AEFI | 1 |
| Musculoskeletal discomfort | Maternal other AEFI | 1 |
| Trigeminal neuralgia | Maternal other AEFI | 1 |
| Phlebitis superficial | Maternal other AEFI | 1 |
| Ear infection | Maternal other AEFI | 1 |
| Tinnitus | Maternal other AEFI | 1 |
| Hypoaesthesia eye | Maternal other AEFI | 1 |
| Swelling of eyelid | Maternal other AEFI | 1 |
| Injection site necrosis | Maternal other AEFI | 1 |
| Lower respiratory tract infection | Maternal other AEFI | 1 |
| Impaired gastric emptying | Maternal other AEFI | 1 |
| Groin pain | Maternal other AEFI | 1 |
| Abdominal discomfort | Maternal other AEFI | 1 |
| Ocular hyperaemia | Maternal other AEFI | 1 |
| Injection site erythema | Maternal other AEFI | 1 |
| Injection site mass | Maternal other AEFI | 1 |
| Injection site warmth | Maternal other AEFI | 1 |
| Mastitis | Maternal other AEFI | 1 |
| Abnormal dreams | Maternal other AEFI | 1 |
| Eye irrigation | Maternal other AEFI | 1 |
| Musculoskeletal chest pain | Maternal other AEFI | 1 |
| Parosmia | Maternal other AEFI | 1 |
| Tension headache | Maternal other AEFI | 1 |
| Herpes zoster | Maternal other AEFI | 1 |
| Fibromyalgia | Maternal other AEFI | 1 |
| Vaccination failure | Maternal other AEFI | 1 |
| Oral herpes | Maternal other AEFI | 1 |
| Embolism | Maternal other AEFI | 1 |
| Nightmare | Maternal other AEFI | 1 |
| Heart rate | Maternal other AEFI | 1 |
| Localised infection | Maternal other AEFI | 1 |
| Skin exfoliation | Maternal other AEFI | 1 |
| Sensitive skin | Maternal other AEFI | 1 |
| Vaccination complication | Maternal other AEFI | 1 |
| Blood glucose increased | Maternal other AEFI | 1 |
| Axillary pain | Maternal other AEFI | 1 |
| Impaired work ability | Maternal other AEFI | 1 |
| Mouth cyst | Maternal other AEFI | 1 |
| Eye irritation | Maternal other AEFI | 1 |
| Dysphonia | Maternal other AEFI | 1 |
| Laryngeal oedema | Maternal other AEFI | 1 |
| Type 1 diabetes mellitus | Maternal other AEFI | 1 |
| Feeling hot | Maternal other AEFI | 1 |
| Nasal discomfort | Maternal other AEFI | 1 |
| Sarcoidosis | Maternal other AEFI | 1 |
| Rash erythematous | Maternal other AEFI | 1 |
| Miliaria | Maternal other AEFI | 1 |
| Discoloured vomit | Maternal other AEFI | 1 |
| Death | Maternal other AEFI | 1 |
| Drug ineffective | Maternal other AEFI | 1 |
| Atrial fibrillation | Maternal other AEFI | 1 |
| Eclampsia | Pregnancy, labour and delivery complications and risk factors (excl abortions and stillbirth) | 1 |
| Subchorionic haematoma | Pregnancy, labour and delivery complications and risk factors (excl abortions and stillbirth) | 1 |
| Cholestasis of pregnancy | Pregnancy, labour and delivery complications and risk factors (excl abortions and stillbirth) | 1 |
| False labour | Pregnancy, labour and delivery complications and risk factors (excl abortions and stillbirth) | 1 |
| Placental insufficiency | Pregnancy, labour and delivery complications and risk factors (excl abortions and stillbirth) | 1 |
| Uterine contractions during pregnancy | Pregnancy, labour and delivery complications and risk factors (excl abortions and stillbirth) | 1 |
| Intrauterine infection | Pregnancy, labour and delivery complications and risk factors (excl abortions and stillbirth) | 1 |
| Imminent abortion | Termination of pregnancy and risk of abortion | 1 |
| Abortion threatened | Termination of pregnancy and risk of abortion | 1 |
| Anembryonic gestation | Termination of pregnancy and risk of abortion | 1 |
| Abortion spontaneous complete | Termination of pregnancy and risk of abortion | 1 |
| Eye swelling | Termination of pregnancy and risk of abortion | 1 |
| Hyperacusis | Termination of pregnancy and risk of abortion | 1 |
| Infection | Termination of pregnancy and risk of abortion | 1 |
| Pelvic pain | Termination of pregnancy and risk of abortion | 1 |
| Pyrexia | Termination of pregnancy and risk of abortion | 1 |
| Menstrual disorder | Termination of pregnancy and risk of abortion | 1 |
| Abortion early | Termination of pregnancy and risk of abortion | 1 |

**D) Janssen vaccine**

| **PTs** | **SMQ** | **N** |
| --- | --- | --- |
| Abortion spontaneous | Termination of pregnancy and risk of abortion | 19 |
| Pyrexia | Maternal other AEFI | 8 |
| Chills | Maternal other AEFI | 7 |
| Thrombosis | Maternal other AEFI | 6 |
| Fatigue | Maternal other AEFI | 6 |
| Nausea | Maternal other AEFI | 4 |
| Headache | Maternal other AEFI | 4 |
| Platelet count decreased | Maternal other AEFI | 4 |
| COVID-19 | Maternal other AEFI | 4 |
| Vaccination failure | Maternal other AEFI | 4 |
| Malaise | Maternal other AEFI | 3 |
| Foetal hypokinesia | Foetal disorders | 3 |
| Dyspnoea | Maternal other AEFI | 2 |
| Pain in extremity | Maternal other AEFI | 2 |
| Ultrasound Doppler abnormal | Maternal other AEFI | 2 |
| Peripheral swelling | Maternal other AEFI | 2 |
| Pulmonary embolism | Maternal other AEFI | 2 |
| Arthralgia | Maternal other AEFI | 2 |
| Condition aggravated | Maternal other AEFI | 2 |
| Myalgia | Maternal other AEFI | 2 |
| Injection site pain | Maternal other AEFI | 2 |
| Syncope | Maternal other AEFI | 2 |
| Pelvic venous thrombosis | Maternal other AEFI | 2 |
| Vaginal haemorrhage | Maternal other AEFI | 2 |
| Caesarean section | Pregnancy, labour and delivery complications and risk factors (excl abortions and stillbirth) | 2 |
| Premature delivery | Pregnancy, labour and delivery complications and risk factors (excl abortions and stillbirth) | 2 |
| Induced labour | Pregnancy, labour and delivery complications and risk factors (excl abortions and stillbirth) | 2 |
| Ultrasound antenatal screen abnormal | Foetal disorders | 2 |
| Cerebrovascular accident | Neonatal other AEFI | 1 |
| Pyrexia | Neonatal other AEFI | 1 |
| Autoimmune disorder | Maternal other AEFI | 1 |
| Pulmonary thrombosis | Maternal other AEFI | 1 |
| Influenza | Maternal other AEFI | 1 |
| Mood altered | Maternal other AEFI | 1 |
| Body temperature increased | Maternal other AEFI | 1 |
| Epidural catheter placement | Maternal other AEFI | 1 |
| Injury | Maternal other AEFI | 1 |
| Procedural haemorrhage | Maternal other AEFI | 1 |
| Swelling face | Maternal other AEFI | 1 |
| Transfusion | Maternal other AEFI | 1 |
| Volume blood decreased | Maternal other AEFI | 1 |
| Impaired work ability | Maternal other AEFI | 1 |
| Localised infection | Maternal other AEFI | 1 |
| Lymphadenitis | Maternal other AEFI | 1 |
| Angiogram | Maternal other AEFI | 1 |
| Chorioretinopathy | Maternal other AEFI | 1 |
| Plasmapheresis | Maternal other AEFI | 1 |
| Platelet transfusion | Maternal other AEFI | 1 |
| Thrombotic thrombocytopenic purpura | Maternal other AEFI | 1 |
| Abnormal uterine bleeding | Maternal other AEFI | 1 |
| Bloody discharge | Maternal other AEFI | 1 |
| Progesterone decreased | Maternal other AEFI | 1 |
| Urinary tract infection | Maternal other AEFI | 1 |
| Urine analysis abnormal | Maternal other AEFI | 1 |
| Pallor | Maternal other AEFI | 1 |
| Uterine haemorrhage | Maternal other AEFI | 1 |
| Dizziness | Maternal other AEFI | 1 |
| Nasopharyngitis | Maternal other AEFI | 1 |
| Anaphylactic shock | Maternal other AEFI | 1 |
| Seizure | Maternal other AEFI | 1 |
| Delivery | Maternal other AEFI | 1 |
| Haemorrhage | Maternal other AEFI | 1 |
| Abdominal pain | Maternal other AEFI | 1 |
| Embolism | Maternal other AEFI | 1 |
| Iron deficiency anaemia | Maternal other AEFI | 1 |
| Pain | Maternal other AEFI | 1 |
| Vomiting | Maternal other AEFI | 1 |
| Chest discomfort | Maternal other AEFI | 1 |
| Chest pain | Maternal other AEFI | 1 |
| Myocarditis | Maternal other AEFI | 1 |
| Troponin increased | Maternal other AEFI | 1 |
| Chest X-ray | Maternal other AEFI | 1 |
| Deep vein thrombosis | Maternal other AEFI | 1 |
| Joint swelling | Maternal other AEFI | 1 |
| Angiogram cerebral abnormal | Maternal other AEFI | 1 |
| Cerebral venous sinus thrombosis | Maternal other AEFI | 1 |
| Magnetic resonance imaging head abnormal | Maternal other AEFI | 1 |
| Postpartum thrombosis | Maternal other AEFI | 1 |
| Facial paresis | Maternal other AEFI | 1 |
| Guillain-Barre syndrome | Maternal other AEFI | 1 |
| Lumbar puncture abnormal | Maternal other AEFI | 1 |
| Neurological symptom | Maternal other AEFI | 1 |
| Paraesthesia | Maternal other AEFI | 1 |
| Rash | Maternal other AEFI | 1 |
| Liver function test abnormal | Maternal other AEFI | 1 |
| Liver injury | Maternal other AEFI | 1 |
| Computerised tomogram abnormal | Maternal other AEFI | 1 |
| Heart rate decreased | Maternal other AEFI | 1 |
| Fibrin D dimer increased | Maternal other AEFI | 1 |
| Immune thrombocytopenia | Maternal other AEFI | 1 |
| SARS-CoV-2 test negative | Maternal other AEFI | 1 |
| Muscle spasms | Maternal other AEFI | 1 |
| Tachycardia | Maternal other AEFI | 1 |
| Thrombocytopenia | Maternal other AEFI | 1 |
| White blood cell count increased | Maternal other AEFI | 1 |
| Stillbirth | Termination of pregnancy and risk of abortion | 1 |
| Labour complication | Pregnancy, labour and delivery complications and risk factors (excl abortions and stillbirth) | 1 |
| Premature labour | Pregnancy, labour and delivery complications and risk factors (excl abortions and stillbirth) | 1 |
| Premature separation of placenta | Pregnancy, labour and delivery complications and risk factors (excl abortions and stillbirth) | 1 |
| Placenta praevia | Pregnancy, labour and delivery complications and risk factors (excl abortions and stillbirth) | 1 |
| HELLP syndrome | Pregnancy, labour and delivery complications and risk factors (excl abortions and stillbirth) | 1 |
| Pre-eclampsia | Pregnancy, labour and delivery complications and risk factors (excl abortions and stillbirth) | 1 |
| Renal aplasia | Congenital, familial and genetic disorders | 1 |
| Foetal heart rate abnormal | Foetal disorders | 1 |
| Ultrasound foetal abnormal | Foetal disorders | 1 |

**E) Mix vaccination**

| **PTs** | **SMQ** | **N** |
| --- | --- | --- |
| Fatigue | Maternal other AEFI | 8 |
| Pain in extremity | Maternal other AEFI | 6 |
| Headache | Maternal other AEFI | 4 |
| Chills | Maternal other AEFI | 4 |
| Abortion spontaneous | Termination of pregnancy and risk of abortion | 4 |
| Oropharyngeal pain | Maternal other AEFI | 2 |
| Pain | Maternal other AEFI | 2 |
| Pyrexia | Maternal other AEFI | 2 |
| Dizziness | Maternal other AEFI | 2 |
| Migraine | Maternal other AEFI | 2 |
| Cough | Maternal other AEFI | 2 |
| Arthralgia | Maternal other AEFI | 2 |
| Malaise | Maternal other AEFI | 2 |
| Nausea | Maternal other AEFI | 2 |
| Myalgia | Maternal other AEFI | 2 |
| Placenta praevia | Pregnancy, labour and delivery complications and risk factors (excl abortions and stillbirth) | 2 |
| Premature delivery | Pregnancy, labour and delivery complications and risk factors (excl abortions and stillbirth) | 2 |
| Faeces discoloured | Neonatal other AEFI | 1 |
| Gastritis | Neonatal other AEFI | 1 |
| Mucous stools | Neonatal other AEFI | 1 |
| Somnolence | Neonatal other AEFI | 1 |
| Adrenal haemorrhage | Neonatal other AEFI | 1 |
| Thrombocytosis | Neonatal other AEFI | 1 |
| Ear pain | Maternal other AEFI | 1 |
| Asthenopia | Maternal other AEFI | 1 |
| Chest pain | Maternal other AEFI | 1 |
| Musculoskeletal stiffness | Maternal other AEFI | 1 |
| Paraesthesia | Maternal other AEFI | 1 |
| Anovulatory cycle | Maternal other AEFI | 1 |
| COVID-19 | Maternal other AEFI | 1 |
| Drug ineffective | Maternal other AEFI | 1 |
| Heavy menstrual bleeding | Maternal other AEFI | 1 |
| Menstruation irregular | Maternal other AEFI | 1 |
| Job dissatisfaction | Maternal other AEFI | 1 |
| Lethargy | Maternal other AEFI | 1 |
| Flatulence | Maternal other AEFI | 1 |
| Influenza like illness | Maternal other AEFI | 1 |
| Vaccination site pain | Maternal other AEFI | 1 |
| Abdominal pain | Maternal other AEFI | 1 |
| Injection site pain | Maternal other AEFI | 1 |
| Foetal death | Termination of pregnancy and risk of abortion | 1 |
| Abortion missed | Termination of pregnancy and risk of abortion | 1 |
| Gestational diabetes | Pregnancy, labour and delivery complications and risk factors (excl abortions and stillbirth) | 1 |
| Placental infarction | Pregnancy, labour and delivery complications and risk factors (excl abortions and stillbirth) | 1 |
| Haemorrhage in pregnancy | Pregnancy, labour and delivery complications and risk factors (excl abortions and stillbirth) | 1 |
| Foetal heart rate abnormal | Foetal disorders | 1 |
| Foetal hypokinesia | Foetal disorders | 1 |
| Exomphalos | Congenital, familial and genetic disorders | 1 |
| Kyphoscoliosis | Congenital, familial and genetic disorders | 1 |
